# Smelling Salts or Smoke and Mirrors? Acute Ammonia Inhalation, Arousal, and Physical Performance: A Systematic Review and Meta-Analysis

**DOI:** 10.3390/brainsci16080849

**Published:** 2026-08-10

**Authors:** Shenghui Liu, Xiaohan Fan, Zhiyuan Tan, Jinfa Gu, Qing Huang, Hansen Li, Lewis J. Macgregor, Hengzhi Deng

**Affiliations:** 1 Faculty of Sports and Exercise Science, Universiti Malaya, Jalan Universiti, Kuala Lumpur 50603, Malaysia; 2 Faculty of Sport and Physical Education, University of Belgrade, Blagoja Parovića 156, 11000 Belgrade, Serbia; 3 School of Health Sciences, Universiti Sains Malaysia, Kubang Kerian 16150, Kelantan, Malaysia; 4 School of Physical Education, Sichuan Agricultural University, Ya’an 625014, China; 5 Centre for Research & Innovation in Sport, University of Stirling, Stirling FK9 4LA, UK

**Keywords:** ammonia inhalants, smelling salts, physical performance, arousal, exercise performance

## Abstract

**Highlights:**

**What are the main findings?**

- Acute ammonia inhalation did not significantly improve overall physical performance but consistently increased subjective activation/arousal.
- Potential performance benefits appeared limited to specific contexts, particularly explosive or repeated high-intensity tasks, and remain supported by low-certainty evidence.

**What are the implications of the main findings?**

- The findings suggest that ammonia inhalation acts primarily as a transient neurobehavioral arousal stimulus rather than a reliable ergogenic aid.
- Future well-controlled studies with standardized protocols are needed to clarify the neural and psychophysiological mechanisms underlying context-dependent performance responses.

**Abstract:**

Background/Objectives: Ammonia inhalants, commonly known as smelling salts, are widely used in sport as an acute arousal strategy, yet their ergogenic value remains uncertain. This systematic review and three-level meta-analysis examined the acute effects of ammonia inhalation on physical performance and selected psychophysiological outcomes. Methods: Controlled human studies involving healthy adults, physically active individuals, or athletes were included when they compared ammonia inhalation with placebo, sham, or no-inhalation control conditions. Physical performance was the primary outcome, with heart rate and selected subjective responses analyzed as exploratory secondary outcomes. Results: Ten studies were included. The primary analysis showed no statistically significant effect of ammonia inhalation on overall physical performance (k = 47, g = 0.11, 95% CI [−0.06, 0.28]). Heart rate and subjective exertion/fatigue were also not significantly affected. In contrast, subjective activation/arousal increased significantly (k = 9, g = 0.73, 95% CI [0.43, 1.04]), although this finding was based on only two studies. Moderator analyses did not show consistent subgroup differences for performance type, training status, or comparator type, whereas sex-based differences were exploratory and based on limited evidence. Sensitivity analyses suggested that physical performance became significantly improved after removal of influential effects, and power/explosive performance showed a possible benefit; however, these findings should be considered preliminary rather than confirmatory. Conclusions: Overall, ammonia inhalation appears to act primarily as a brief arousal stimulus rather than a consistent ergogenic aid. Potential benefits may depend on task demands, with explosive or repeated high-intensity tasks appearing more plausible targets than maximal strength or precision-based skills. Current evidence remains limited by small samples, heterogeneous protocols, and low certainty.

## 1. Introduction

Ammonia inhalants, commonly known as smelling salts, have long been used in sport and exercise settings as an acute stimulant strategy. Although originally introduced in medical contexts to counteract faintness, they are now frequently used by athletes before maximal or high-intensity efforts, particularly in strength, power, and collision-based sports [1,2]. Their practical appeal lies in the rapid and easily administered nature of the intervention, with athletes often using them to increase alertness, perceived readiness, and psychological activation immediately before performance attempts. However, compared with more established ergogenic aids, the effects of ammonia inhalants on physical performance remain relatively underexplored.

The proposed effects of ammonia inhalation are thought to be primarily neurophysiological and psychophysiological rather than metabolic. Ammonia vapor stimulates the nasal and respiratory mucosa, producing a strong irritant response that may transiently increase respiratory drive, sympathetic activation, heart rate, and subjective arousal [2]. Such short-term stimulation may theoretically benefit tasks requiring rapid force production, explosive effort, or repeated high-intensity output. In addition, heightened perceived alertness or readiness may influence effort allocation during demanding exercise.

Despite this plausible rationale, empirical findings remain inconsistent. Some studies have reported potential benefits for explosive or repeated high-intensity performance [3,4], whereas others have observed trivial or no effects on maximal strength or related performance outcomes [5,6,7]. Therefore, the extent to which ammonia inhalation provides a meaningful ergogenic effect across different physical performance domains, rather than merely a transient arousal response, remains unclear.

Several reviews have summarized the current literature on ammonia inhalants in humans and sport settings [1,2]. A recent systematic review also highlighted the limited and inconsistent evidence regarding ammonia inhalation for enhancing physical performance and exercise tolerance [8]. However, these syntheses have remained primarily qualitative and have not provided pooled estimates of effect magnitude or precision. As a result, although ammonia inhalants are widely used in practice, the overall direction, magnitude, and practical relevance of their effects on physical performance remain uncertain.

A quantitative synthesis is therefore needed to clarify the current evidence, particularly because individual studies are generally small and have used varied exercise tasks, comparator conditions, and outcome measures. Accordingly, this systematic review and three-level meta-analysis aimed to examine the acute effects of ammonia inhalation on physical performance in healthy adults, physically active individuals, and athletes. Secondary aims were to evaluate related psychophysiological outcomes and to explore whether effects differ across exercise modalities and participant characteristics. By providing a quantitative estimate of the current evidence, this review may help determine whether ammonia inhalation represents a practically relevant ergogenic aid or a context-dependent arousal strategy.

## 2. Methods

This systematic review and meta-analysis was pre-registered on the Open Science Framework (OSF) on 29 May 2026 (Registration: osf.io/hxuve) and conducted in accordance with the PRISMA 2020 guidelines [9]. The completed PRISMA checklist is available in Electronic Supplementary Material S1.

### 2.1. Eligibility Criteria

This review followed the PICOS framework: (1) participants—healthy adults (≥18 years), including physically active individuals and athletes; (2) intervention—acute ammonia inhalation, including ammonia inhalants, smelling salts, or aromatic ammonia; (3) comparison—placebo, sham, or control condition; and (4) outcomes—physical performance as the primary outcome, with selected psychophysiological responses, including heart rate, and subjective responses related to exertion/fatigue and activation/arousal, as secondary outcomes.

Studies were eligible for inclusion if they met all of the following criteria: (1) original controlled human experimental studies; (2) examined the acute effects of ammonia inhalation before or during exercise or sport-related tasks; (3) involved healthy adult participants, physically active individuals, or athletes; (4) included a placebo, sham, or control comparison; and (5) reported at least one physical performance outcome or relevant psychophysiological outcome with sufficient data for effect size calculation.

Studies were excluded if they met any of the following criteria: (1) involved clinical populations, injured participants, or medical resuscitation contexts; (2) did not examine ammonia inhalation, smelling salts, aromatic ammonia, or a comparable ammonia-based inhalant; (3) lacked a comparator condition; (4) did not assess exercise, physical performance or sport-related outcomes; (5) were reviews, editorials, commentaries, case reports, animal studies, or in vitro studies; or (6) lacked sufficient methodological or statistical information for eligibility assessment or effect size calculation.

Acute exposure was defined a priori as ammonia inhalation administered before and/or during a single exercise session or sport-related task, with outcomes assessed within the same experimental visit. The primary objective of this review was to evaluate the effect of ammonia inhalation on objective physical performance. Therefore, performance outcomes were prioritized for quantitative synthesis, while secondary psychophysiological outcomes were extracted and analyzed where available. Studies reporting only non-exercise or medical outcomes were excluded to maintain alignment with the review objective.

### 2.2. Data Sources and Search Strategy

The final systematic search was conducted on 29 May 2026, across PubMed, Web of Science, Cochrane Library, Scopus, and SPORTDiscus. The following Boolean search strategies were applied: (“ammonia inhal*” OR “ammonia inhalant*” OR “smelling salt*” OR “aromatic ammonia”) AND (exercise OR sport OR athlete* OR performance OR strength OR power OR sprint* OR endurance OR fatigue OR arousal OR alertness).

No date or filter restrictions were applied.

### 2.3. Data Extraction

All records were imported into Microsoft Excel and EndNote 21 for de-duplication and management. Two independent reviewers screened titles, abstracts, and full texts for eligibility, with disagreements resolved through discussion and consensus. For each eligible study, extracted data included sample size, participant characteristics such as age, sex, training status, and sport or exercise background, study design, intervention and comparator details, exercise modality and protocol, outcome measures, and statistical data required for effect size calculation.

Intervention details included the type of ammonia inhalant, product description or concentration where reported, distance from the nose, exposure duration, number of inhalations, and timing of inhalation relative to the exercise task. Comparator details included placebo, or control conditions.

The primary outcome was physical performance, defined as the direct performance endpoint reported in each study, including strength, power, sprint or repeated-sprint performance, anaerobic performance, repetitions to failure, and sport- or exercise-specific performance outcomes. Secondary outcomes included heart rate and selected subjective responses. Subjective responses were grouped into two domains: subjective exertion/fatigue, including rating of perceived exertion (RPE); and subjective activation/arousal, including alertness and psyched-up energy. For secondary outcomes, values were preferentially extracted under the same condition as the corresponding performance outcome; when repeated measurements were available, the value at the final exercise time point or the closest post-exercise assessment was extracted to improve comparability across studies. Subjective measures collected before ammonia or comparator administration were not extracted as ammonia-related outcomes.

When data were not reported numerically, authors were contacted or WebPlotDigitizer (v4.8) was used for extraction [10].

### 2.4. Quality and Risk of Bias Assessment

Methodological quality was assessed using a modified version of the Physiotherapy Evidence Database (PEDro) scale, with an additional item evaluating whether the effectiveness of blinding to the placebo or sham condition was assessed [11]. The total score ranged from 0 to 11, with studies categorized as excellent (10–11), good (7–9), fair (5–6), or poor (<5). Two reviewers independently evaluated all included studies, and discrepancies were resolved through discussion or adjudication by a third reviewer when consensus could not be reached.

In parallel, risk of bias was evaluated using the Cochrane Risk of Bias 2 (RoB 2) tool. For crossover trials, the RoB 2 version adapted for crossover designs was applied, including the additional domain assessing bias arising from period and carryover effects [12]. The evaluated domains included the randomization process, deviations from intended interventions, missing outcome data, outcome measurement, selection of the reported result, and period or carryover effects where applicable. Assessments were performed independently by two reviewers, with disagreements resolved in the same manner.

### 2.5. Statistical Analysis

#### 2.5.1. Effect Size Calculation and Data Synthesis

All effect size calculations adhered to the Cochrane Handbook for Systematic Reviews of Interventions (Version 6.5, 2024) [13]. Given the expected small sample sizes of included studies, Hedges’ g was used to estimate standardized mean differences between ammonia inhalation and comparator conditions, with correction for small-sample bias [14].

As the eligible studies were expected to primarily use within-subject crossover designs, effect sizes were calculated by accounting for the paired-sample structure. When available, within-participant correlations were used to derive the variance of standardized mean differences. If pre–post data were reported, change scores were used where appropriate.

Because most studies were unlikely to report within-participant correlations, a correlation coefficient of r = 0.50 was assumed for the primary analysis [15], Sensitivity analyses were conducted using r = 0.20 (lower bound) and r = 0.80 (upper bound) to examine the robustness of the findings [16].

Effect sizes were (g) interpreted using standard thresholds: trivial (<0.2), small (0.2–0.5), medium (0.5–0.8), and large (>0.8). Detailed computational formulas and step-by-step procedures are provided in Supplementary Material S2.

#### 2.5.2. Three-Level Meta-Analysis and Heterogeneity

To account for dependency arising from multiple outcomes within the same study, three-level meta-analytic models were performed using the *metafor* package in RStudio (version 2024.09.1, Build 394) with restricted maximum likelihood (REML) estimation [17,18,19]. Variance was partitioned into sampling variance (level 1), within-study variance (level 2), and between-study variance (level 3) [20]. All inferential statistics were adjusted using cluster-robust variance estimation with CR2 small-sample correction and Satterthwaite degrees of freedom, where sufficient independent study clusters were available, with *p*-values and 95% confidence intervals (CI) reported from the CR2-adjusted models [21]. Model robustness was additionally examined by comparing results obtained using maximum likelihood (ML) estimation.

Heterogeneity was quantified using I^2^ statistics and interpreted as low (0–25%), moderate (25–49%), substantial (50–74%), or considerable (≥75%) [15,22]. Prediction intervals (PI) were calculated to estimate the expected range of true effects in comparable future studies [23,24]. Power analyses were conducted to assess the risk of Type II error [25].

#### 2.5.3. Moderators and Subgroup Analysis

To explore potential sources of heterogeneity, subgroup analyses were conducted for the primary outcome, physical performance. Given the limited number of included studies and effect sizes, these analyses were considered exploratory and interpreted cautiously. The following moderators were examined: participant sex, training status, physical performance domain, and comparator type.

Participant sex was categorized as male-only, female-only, or mixed samples. Training status was classified as trained or untrained according to established participant categorization frameworks [26], with recreationally active, trained/developmental, well-trained/national-level, and elite/international-level participants classified as trained.

Physical performance outcomes were grouped into three domains: strength performance, power/explosive performance, and sport- or task-specific performance. Strength performance included maximal strength, maximal force, and repetitions to failure. Power/explosive performance included jump performance, Wingate power outcomes, movement velocity, power output, and rate of force development. Sport- or task-specific performance included outcomes reflecting performance in a specific sport or applied task, such as putting performance, shooting accuracy, or rifle disassembly/reassembly time.

Comparator type was categorized as sham inhalation control or no-inhalation control. Sham inhalation included placebo or sham conditions involving an inhalation procedure, such as water, menthol, or Vick’s VapoRub, whereas no-inhalation control referred to conditions without an active or sham inhalant.

Other potential moderators, including ammonia dose, product form, administration distance, exposure duration, and timing of administration, were not further examined because of substantial heterogeneity and insufficient reporting across studies. All subgroup analyses used the same three-level meta-analytic framework as the main analysis, with cluster-robust variance estimation using the CR2 adjustment to account for dependence among effect sizes within studies [21]. All visualizations were generated using *ggplot2* and *orchaRd* packages [27].

#### 2.5.4. Publication Bias and Sensitivity Analyses

Contour-enhanced funnel plots [28] and Egger’s regression tests [29] were used exploratorily to assess small-study effects and potential publication bias for primary and secondary outcomes, as well as eligible subgroup analyses, when at least 10 effect sizes were available (k ≥ 10) [30].

Sensitivity analyses included: (1) varying the assumed within-subject correlation coefficient (r), using values of 0.20 and 0.80; (2) leave-one-out analyses; and (3) exclusion of influential outliers identified using Cook’s distance and studentized residuals. Among these, outlier exclusion was applied not only to the overall models but also across all moderator and meta-regression analyses to ensure robustness of subgroup inferences, whereas the remaining sensitivity checks were conducted for the main pooled effects.

### 2.6. Certainty of the Evidence

The certainty of evidence was assessed using the Grading of Recommendations Assessment, Development, and Evaluation (GRADE) framework, considering risk of bias, inconsistency, indirectness, imprecision, and publication bias [31]. Overall certainty was classified as high, moderate, low, or very low. GRADE assessments were conducted independently by one reviewer (D.H.Z) and verified by a second (F.X.H), with any discrepancies resolved through discussion to achieve consensus.

## 3. Results

### 3.1. Studies Retrieved

**Figure 1:**
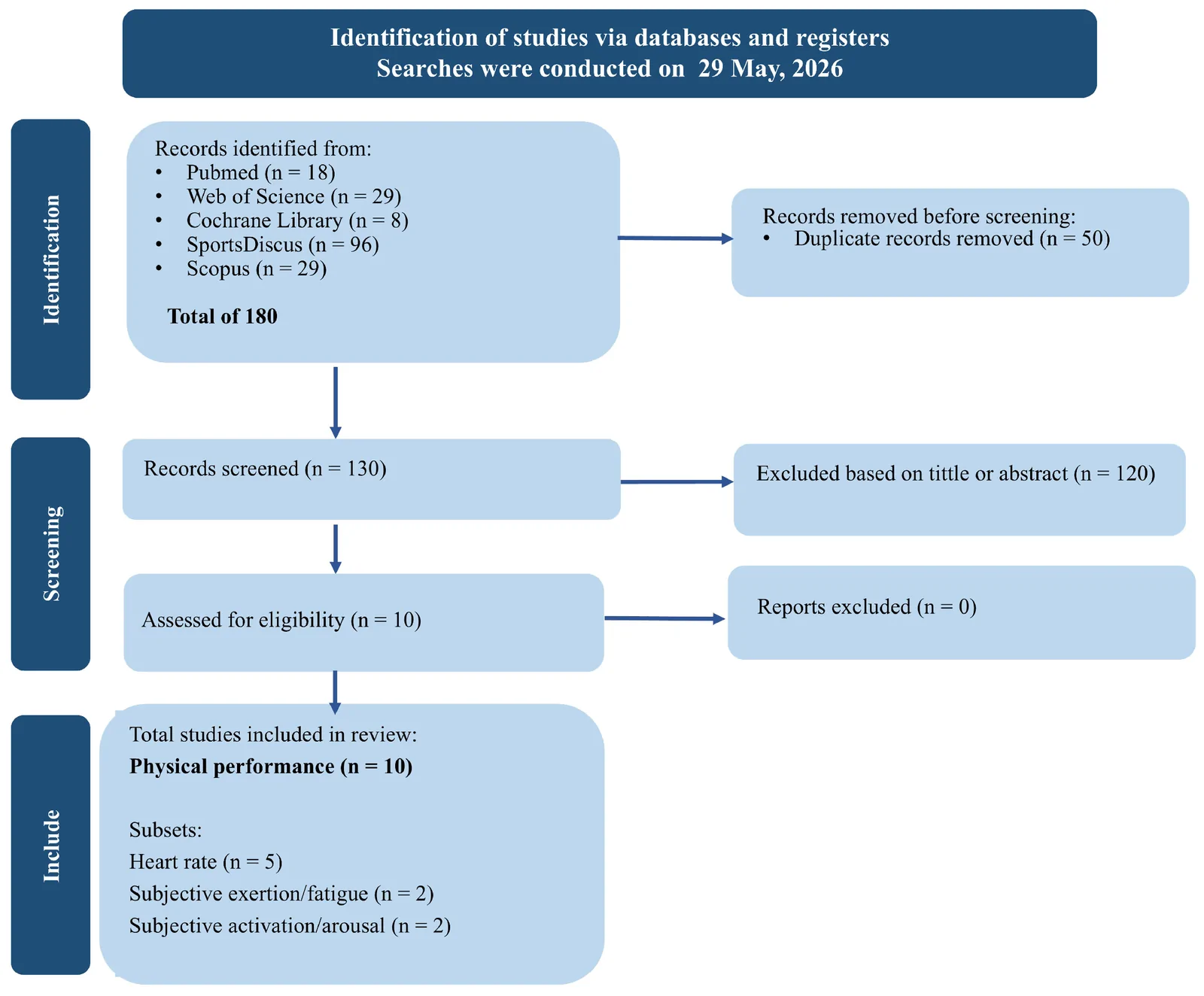
PRISMA flow diagram for included and excluded studies.

The initial database search identified 180 records. After duplicate removal and title, abstract, and full-text screening, 10 studies met the inclusion criteria and evaluated physical performance. These studies contributed a total of 47 effect size estimates (k = 47). In addition, five studies (k = 12) reported heart rate, two studies (k = 9) reported subjective exertion/fatigue, and two studies (k = 9) reported subjective activation/arousal (Figure 1).

### 3.2. Characteristics of Included Studies

Across the 10 included studies, a total of 163 participants were analyzed, with sample sizes ranging from 9 to 25 participants. Most participants were men (141 men and 22 women). Eight studies recruited male-only samples [3,5,6,32,33,34,35,36], one study recruited a female-only sample [4], and one study recruited a mixed-sex sample with data available separately by sex [7].

Regarding training status, only one study was classified as involving untrained participants [34], whereas the remaining nine studies included trained participants, ranging from recreationally active individuals to resistance-trained participants, military cadets, and professional athletes. Physical performance outcomes were grouped into three domains: strength performance, power/explosive performance, and sport- or task-specific performance. Strength outcomes included maximal force, 1-RM performance, and repetitions to failure (6 studies, k = 13). Power/explosive outcomes included countermovement jump performance, Wingate power outcomes, movement velocity, power output, and rate of force development (6 studies, k = 23). Sport- or task-specific outcomes included golf putting, shooting accuracy, and rifle disassembly/reassembly performance (2 studies, k = 11).

Ammonia inhalation protocols varied across studies, most commonly involving commercially available ammonia capsules or ampules, with reported doses typically around 0.3–0.33 mL and ammonia concentrations of approximately 15% where specified. Administration timing also differed, with ammonia generally inhaled shortly before each performance attempt, ranging from immediately before testing to approximately 10–30 s before the task. Comparator conditions were categorized as sham inhalation or no-inhalation controls. Six studies included a sham inhalation comparator, such as water, menthol, or Vick’s VapoRub [4,5,6,7,34,35], while five studies included a no-inhalation control condition [3,5,32,33,36]. One study included both sham inhalation and no-inhalation control conditions [5]. For more details, please refer to Table 1.

### 3.3. Primary Analysis

The primary meta-analysis showed a small, non-significant effect of ammonia inhalation on overall physical performance (k = 47, n = 10, g = 0.11, 95% CI [−0.06, 0.28], I^2^ = 31%, PI [−0.27, 0.49], *p* = 0.17, power = 37%), with the certainty of evidence rated as very low.

**Figure 2:**
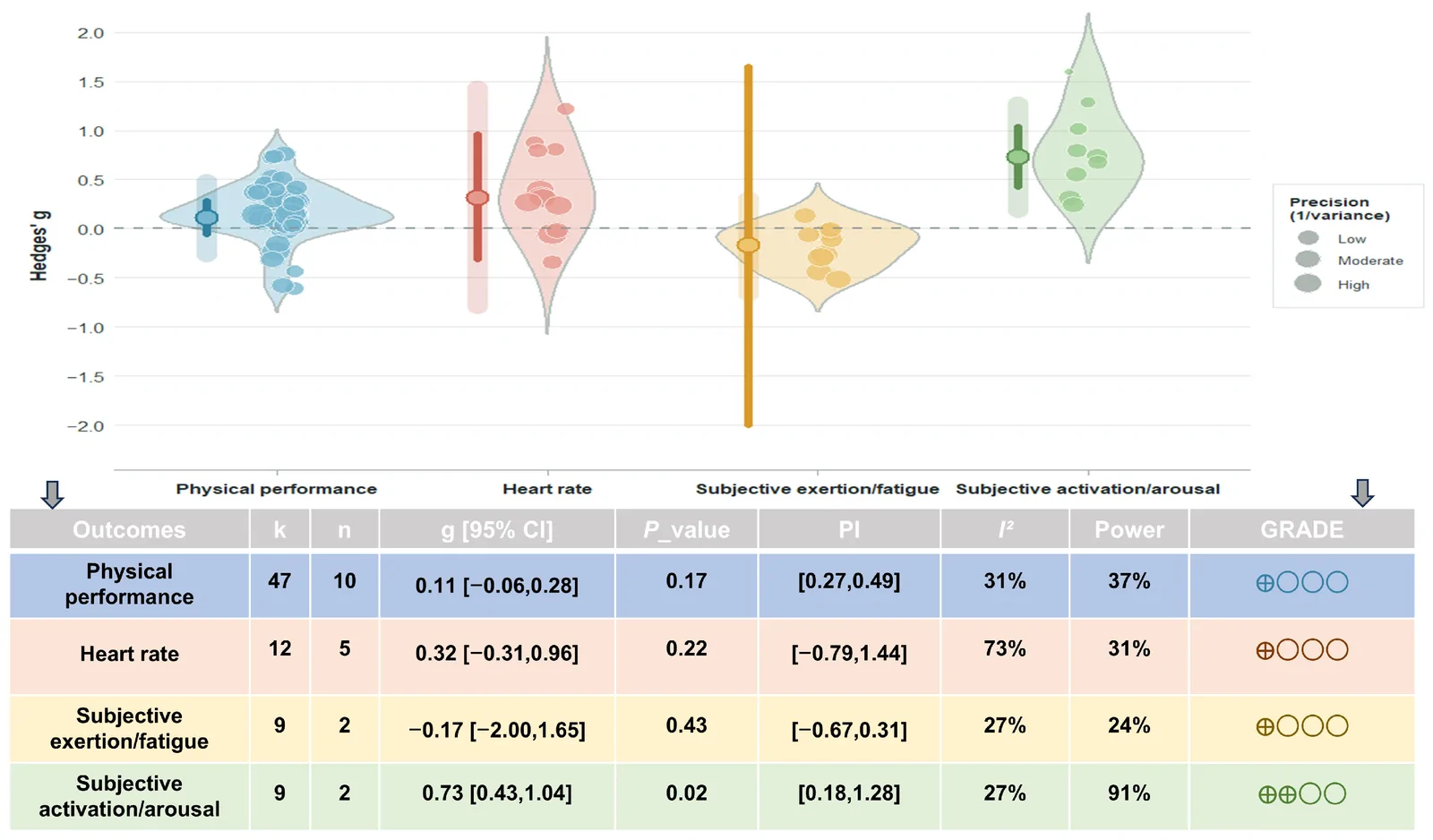
Primary pooled effect sizes for acute ammonia inhalation on physical performance, perceptual, and physiological outcomes. Notes: K, the total number of effects included in the pooled effect size; Hedges’ g, the effect size indicators used in the pooled; 95%CI, 95% confidence interval after CR2 small-sample correction; PI, prediction interval; *p*-value, statistically significant *p* values for pooled results after CR2 small-sample correction; I^2^, quantitative indicators of heterogeneity; Power, exploratory post hoc statistical power estimate for pooled effect size; GRADE, grading of recommendations assessment, development, and evaluation, a system for evaluating the quality of evidence and strength of recommendations.

For exploratory secondary outcomes, heart rate showed a small-to-moderate, non-significant increase (k = 12, n = 5, g = 0.32, 95% CI [−0.31, 0.96], I^2^ = 73%, PI [−0.79, 1.44], *p* = 0.22, power = 31%), with very low certainty of evidence. Subjective exertion/fatigue was not significantly affected (k = 9, n = 2, g = −0.17, 95% CI [−2.00, 1.65], I^2^ = 27%, PI [−0.67, 0.31], *p* = 0.43, power = 24%), also with very low certainty of evidence. Subjective activation/arousal showed a statistically significant positive effect (k = 9, n = 2, g = 0.73, 95% CI [0.43, 1.04], I^2^ = 27%, PI [0.18, 1.28], *p* = 0.02, power = 91%); however, this estimate was based on only two studies and the certainty of evidence was rated as low (Figure 2; see also Supplementary Material S3 for the conventional forest plot).

### 3.4. Moderator Analysis

To explore potential sources of heterogeneity and further interpret the overall findings, moderator analyses were conducted for physical performance. Given the small number of included studies within several strata, these analyses were considered supplementary to the primary analysis.

When stratified by sex, neither male-only samples (k = 38, g = 0.08, 95% CI [−0.06, 0.23], I^2^ = 14%, PI [−0.16, 0.33], *p* = 0.21; GRADE: Very low) nor female samples (k = 9, g = 0.40, 95% CI [−1.77, 2.56], I^2^ = 30%, PI [0.05, 0.75], *p* = 0.26; GRADE: Low) showed a statistically significant effect. The between-subgroup test suggested a potential difference by sex (*p* = 0.04), although this finding should be interpreted with caution because female data were derived from limited evidence.

By training status, trained participants showed a small, non-significant effect (k = 45, g = 0.15, 95% CI [−0.02, 0.31], I^2^ = 25%, PI [−0.18, 0.47], *p* = 0.07; GRADE: Very low). The untrained subgroup was based on only two effect sizes and also showed no significant effect (k = 2, g = −0.23, 95% CI [−0.72, 0.27], PI [−0.81, 0.35], *p* = 0.11; GRADE: Very low). No significant subgroup difference was observed by training status (*p* = 0.15).

For performance type, no statistically significant effects were observed for strength performance (k = 13, g = 0.05, 95% CI [−0.13, 0.23], I^2^ = 0%, PI [−0.35, 0.45], *p* = 0.48; GRADE: Low), power/explosive performance (k = 23, g = 0.16, 95% CI [−0.13, 0.45], I^2^ = 43%, PI [−0.23, 0.55], *p* = 0.21; GRADE: Very low), or sport- or task-specific performance (k = 11, g = 0.10, 95% CI [−1.20, 1.40], I^2^ = 76%, PI [−0.34, 0.53], *p* = 0.70; GRADE: Very low). The between-subgroup test was not significant (*p* = 0.54).

Comparator type also did not appear to modify the effect. Similar non-significant effects were observed for sham inhalation controls (k = 19, g = 0.11, 95% CI [−0.15, 0.37], I^2^ = 38%, PI [−0.32, 0.54], *p* = 0.34; GRADE: Very low) and no-inhalation controls (k = 28, g = 0.10, 95% CI [−0.16, 0.36], I^2^ = 0%, PI [−0.34, 0.54], *p* = 0.32; GRADE: Low), with no evidence of a subgroup difference (*p* = 0.96).

**Figure 3:**
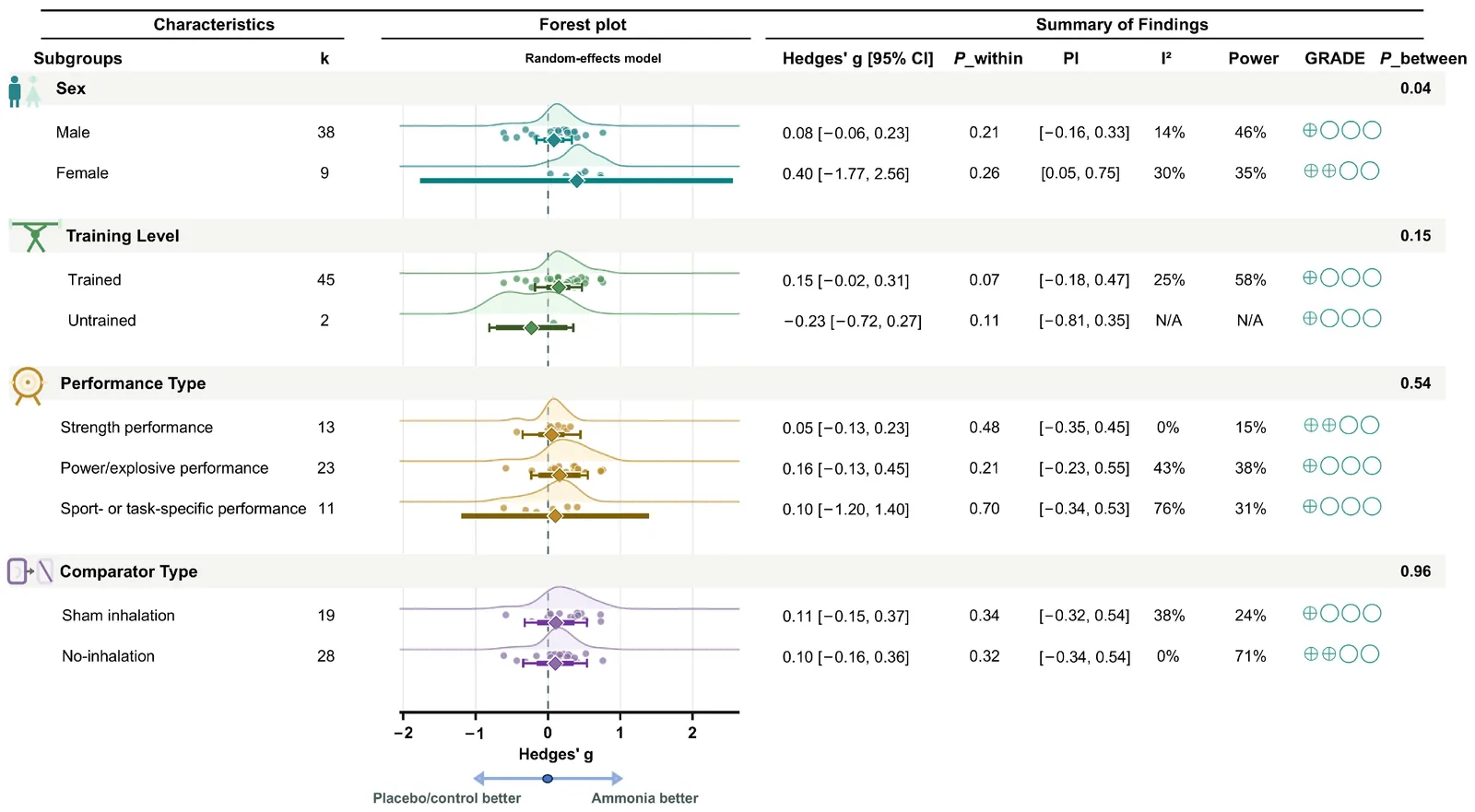
Moderator analysis for the primary physical performance outcome. Notes: K, the total number of effects included in the pooled effect size; Hedges’ g, the effect size indicators used in the pooled; 95%CI, 95% confidence interval; PI, prediction interval after CR2 small-sample correction; P_between_, CR2-adjusted *p* value for subgroup differences; P_within_, CR2-adjusted *p* value for the pooled effect within each subgroup; I^2^, quantitative indicators of heterogeneity; Power, exploratory post hoc statistical power estimate for pooled effect size; GRADE, grading of recommendations assessment, development, and evaluation, a system for evaluating the quality of evidence and strength of recommendations.

For more details, please refer to Figure 3.

### 3.5. Risk of Bias and Quality of Methods

Across studies, none was judged to be at overall low risk of bias; nine were rated as having some concerns and one as high risk [36]. At the domain level, missing outcome data was consistently judged as low risk. Period and carryover effects were also generally rated as low risk, with the exception of three studies that employed same-session crossover procedures with relatively short intervals between ammonia and control conditions [3,32,36]. In contrast, the randomization process was mostly rated as some concerns, as only one study clearly reported adequate randomization/allocation procedures [7], while the remainder provided insufficient detail. Deviations from intended interventions represented the main source of higher risk, largely due to incomplete or unsuccessful blinding, especially where ammonia was compared with water or no-inhalant controls. Measurement of outcomes was generally low risk because performance outcomes were mostly objective, with only limited concern in one study [36]. Selection of the reported result was rated as some concerns in all studies, primarily because none provided a sufficiently detailed prespecified protocol or trial registration for the reported analyses. Overall, the evidence base shows a predominantly moderate risk-of-bias profile, driven mainly by unclear randomization reporting, blinding limitations, and lack of prespecification, rather than widespread missing data or outcome measurement problems (Supplementary Material S4).

Visual inspection of contour-enhanced funnel plots, together with Egger’s regression tests, did not indicate statistically significant evidence of funnel-plot asymmetry or small-study effects for the primary physical performance outcome or eligible secondary outcomes. In subgroup and moderator analyses, Egger’s regression was conducted only for strata comprising at least 10 effect sizes (k ≥ 10), and no statistically significant asymmetry was detected in any eligible subgroup.

However, these findings should be interpreted cautiously because several outcomes and subgroup categories included a small number of studies and effect sizes, limiting the power of funnel-plot-based methods and Egger’s regression tests to detect small-study effects. Detailed results are provided in Supplementary Materials S5 and S6.

The mean modified PEDro score was 6.6, indicating fair-to-good methodological quality across included studies. More information is provided in Supplementary Material S7. In addition, the certainty of evidence for each outcome was evaluated using the GRADE approach. Overall, the certainty of evidence ranged from low to very low across outcomes, mainly due to risk of bias, inconsistency, imprecision, and sparse evidence in several analyses. Full details are provided in Supplementary Material S8.

### 3.6. Sensitivity Analysis

#### 3.6.1. Sensitivity Analysis for Primary Effect

Sensitivity analyses were conducted to examine the robustness of the primary and secondary outcome estimates. For physical performance, the pooled effect remained non-significant when assuming higher or lower within-study correlations and when using maximum likelihood estimation. However, after primarily excluding outlying effects from two studies [32,34], the pooled estimate became statistically significant (k = 42, g = 0.21, 95% CI [0.07, 0.35], *p* = 0.01), with heterogeneity reduced to 12%.

For heart rate, sensitivity analyses produced similar non-significant estimates across alternative assumptions, and removal of outliers did not materially change the result (k = 10, g = 0.30, 95% CI [−0.38, 0.98], *p* = 0.19). Leave-one-out analyses for physical performance and heart rate did not identify any single study that substantially altered the pooled estimate. Leave-one-out analyses were not performed for subjective exertion/fatigue or subjective activation/arousal because each outcome was informed by only two studies.

For subjective exertion/fatigue, results remained non-significant across all sensitivity analyses. Subjective activation/arousal remained statistically significant under alternative correlation assumptions and maximum likelihood estimation, although no outlier-exclusion analysis was performed for this outcome. For more details, please refer to Supplementary Materials S9 and S10.

#### 3.6.2. Sensitivity Analysis for Moderator Effect

Sensitivity analyses for moderator effects showed some changes after exclusion of influential observations. In the sex subgroup analysis, both male (k = 34, g = 0.15, 95% CI [0.04, 0.26], *p* = 0.02) and female samples (k = 8, g = 0.47, 95% CI [0.34, 0.60], *p* = 0.01) showed statistically significant effects, with a significant between-subgroup difference (*p* = 0.01). For training status, only the trained subgroup retained sufficient data, and the effect remained non-significant (k = 45, g = 0.14, 95% CI [−0.02, 0.31], *p* = 0.07).

For performance type, a significant effect emerged for power/explosive performance (k = 20, g = 0.25, 95% CI [0.02, 0.49], *p* = 0.04), whereas strength performance and sport- or task-specific performance remained non-significant. The between-subgroup test was not significant (*p* = 0.27). For comparator type, effects remained non-significant for both sham inhalation and no-inhalation controls, with no evidence of a subgroup difference (*p* = 0.98). Overall, these sensitivity analyses suggest that some subgroup estimates were sensitive to influential observations and are therefore best regarded as supplementary evidence on the stability of the findings rather than as primary evidence of subgroup-specific effects (Supplementary Material S11).

## 4. Discussion

### 4.1. Main Findings and Interpretation

The present systematic review and three-level meta-analysis provides a quantitative synthesis of the acute effects of ammonia inhalation on physical performance and selected psychophysiological outcomes. In the primary analysis, ammonia inhalation did not significantly improve overall physical performance, although the pooled estimate was small and positive. This finding suggests that ammonia inhalation should not be interpreted as a broadly effective ergogenic aid. Rather, its effects appear to be context-dependent, with potential benefits more likely when the acute increase in arousal is compatible with the demands of the task. Accordingly, the primary finding should be viewed as evidence against a general performance-enhancing effect, while leaving open the possibility of task-specific responses that require further confirmation.

From a mechanistic perspective, ammonia inhalants are unlikely to enhance performance through metabolic pathways. Their primary effect is more plausibly related to strong nasal and respiratory irritation, which activates trigeminal chemosensory pathways and evokes an acute respiratory, sympathetic, and perceptual response [1,37]. This mechanism is consistent with the present finding that subjective activation/arousal increased significantly, although this outcome was based on only two studies. By contrast, heart rate and subjective exertion/fatigue did not show clear or consistent effects. In other words, ammonia inhalation may alter the psychological and sensory state immediately before performance, but this does not necessarily translate into improved physical output. Thus, the proposed arousal-based explanation remains plausible, but the current evidence points to this pathway as a plausible link between ammonia inhalation and acute subjective activation, rather than demonstrating it directly, since the relevant physiological, attentional, and neural pathways were not directly evaluated in most included studies.

The sensitivity analysis provides an important nuance. After the exclusion of outlying effects [32], including those from Ahn and Ko (2022), the pooled effect for physical performance became statistically significant, indicating that the overall estimate was sensitive to specific task types. Ahn and Ko examined 3 m golf putting success in professional golfers, a fine-motor and precision-based task requiring postural stability, attentional control, and calm execution. In such contexts, an intense irritant stimulus may be poorly matched to task demands and could plausibly disrupt rather than enhance performance. This interpretation is consistent with classical arousal-performance theory, which suggests that high arousal may impair complex or precision-based skills [38], and with attentional theories proposing that arousal or pressure can narrow cue utilization and disrupt skilled motor control [39,40]. A similar pattern was observed in Maleček et al. (2023), where ammonia inhalation did not improve shooting accuracy, another task requiring steadiness and precision [36]. Therefore, the influence of Ahn and Ko (2022) may not simply reflect statistical noise [32], but may indicate that ammonia inhalation is less suitable for calm, accuracy-dependent sport skills.

Consistent with this task-demand interpretation, although the primary moderator analysis did not show a clear difference across performance types, the sensitivity analysis suggested a possible signal for power/explosive performance. Although maximal strength and explosive performance share common neuromuscular determinants, they differ in the time scale over which force must be expressed. Explosive outcomes, particularly rate of force development and short-duration power tasks, depend heavily on the rapid rise in neural drive and the ability to generate force within the early phase of contraction [41,42]. A brief increase in alertness or readiness may therefore be more likely to influence the intent and rapid initiation of force production, especially when the task is performed immediately after inhalation. In contrast, maximal strength outcomes, such as 1-RM or peak isometric force, allow more time to approach maximal force and are strongly constrained by broader morphological, technical, and task-specific factors [43,44]. This may explain why ammonia inhalation appears more promising for explosive or repeated high-intensity outcomes than for maximal strength performance. This interpretation aligns with Bartolomei et al. (2018), who reported improved peak rate of force development [5], and Rogers et al. (2023), who observed greater Wingate power alongside increased alertness and psyched-up energy [4]. However, because this subgroup effect emerged in sensitivity analysis and the certainty of evidence was low to very low, it should be viewed as a preliminary task-specific signal rather than a confirmed effect.

The sex-based moderator findings also warrant cautious consideration. The between-subgroup test suggested a potential difference by sex, and the female subgroup showed a larger point estimate than the male subgroup. This pattern may partly reflect the influence of Rogers et al. (2023), in which physically active women demonstrated improved repeated high-intensity cycling performance and increased subjective activation after ammonia inhalation [4]. However, the female evidence base was small and derived from limited studies and effect sizes, making it difficult to determine whether the apparent difference reflects a true sex-specific response, differences in task type, sampling variability, or study-level confounding. At present, the data are insufficient to support firm conclusions regarding sex differences.

Training status also did not provide a reliable explanation for the overall findings. Most included studies involved trained or physically active participants, whereas the untrained subgroup was informed by very limited evidence. Therefore, although trained participants showed a small positive estimate, and untrained participants did not, this comparison is underpowered and likely confounded by differences in task selection and study design. Future work should avoid treating training status as a simple binary construct and should more precisely characterize resistance-training history, sport background, competitive level, and familiarity with ammonia inhalants.

Comparator design is another important issue. In the present analysis, effects were similar when ammonia inhalation was compared with sham inhalation and no-inhalation controls, and the between-subgroup difference was not significant. This pattern suggests that the observed effects are unlikely to be explained solely by the behavioral ritual of inhaling something or by a generic placebo-like expectation. However, this interpretation is limited by the fact that ammonia has a distinctive odor and produces an immediate irritant response, making effective blinding difficult even when water, menthol, or Vick’s VapoRub is used as a sham comparator. As noted by Maleček et al. (2023), participants can likely distinguish ammonia from placebo because of its strong sensory characteristics [36]. Thus, expectancy effects cannot be ruled out, although comparator design alone does not appear to fully explain the observed pattern of results.

A further consideration is the substantial heterogeneity in administration protocols. Across studies, ammonia inhalants differed in form, concentration, dose, distance from the nose, exposure duration, timing relative to performance, and number of inhalations. Some studies used capsules or ampules of approximately 0.3–0.33 mL, whereas others used ammonia crystals or less clearly described products. Timing also varied from immediate inhalation to 10–30 s before performance attempts, and in some protocols ammonia was administered repeatedly before each effort. This issue is important because any arousal response is likely brief and may not peak at the exact moment of performance. Perry et al. (2016) observed that heart rate increased significantly 15 s after ammonia inhalation [3], whereas responses immediately after inhalation and at later time points were less pronounced, suggesting that the onset and duration of the physiological response may be both delayed and short-lived. If a task requires a longer setup, bracing routine, or technical preparation, the peak perceptual or physiological response may not coincide with the actual performance attempt. Overall, the current literature remains too heterogeneous to identify an optimal administration strategy.

Taken together, the present findings are best interpreted through a task-demand framework. Ammonia inhalation appears to create a short-lived activation state, but the performance relevance of this state depends on whether increased arousal supports or disrupts the specific motor task. This framework helps reconcile the mixed findings across explosive, strength, repeated high-intensity, and precision-based outcomes without assuming a uniform ergogenic effect. It also highlights that “physical performance” is not a single construct: the same acute stimulus may have different consequences depending on the required balance between force, speed, attention, precision, and timing.

### 4.2. Strengths and Limitations

This review has several strengths. It is among the first quantitative syntheses to examine ammonia inhalation and physical performance using a three-level meta-analytic model, allowing dependent effect sizes within studies to be retained. It also separated physical performance into strength, power/explosive, and sport- or task-specific domains, which is important given the distinct physiological and psychological requirements of these outcomes. In addition, the review considered comparator type, sex, and training status as moderators and evaluated certainty of evidence using GRADE.

Several limitations should also be acknowledged. The evidence base was small, and many secondary and subgroup analyses were based on few studies or effect sizes. Several statistically significant findings appeared only in sensitivity analyses and should therefore be interpreted cautiously. Risk of bias was also a concern, particularly due to incomplete reporting of randomization, limited preregistration, and challenges with blinding. Blinding is especially difficult in ammonia inhalation studies because ammonia has a distinctive odor and produces immediate nasal and respiratory irritation. As a result, participants may have been able to identify the active condition even when sham comparators were used, which could have influenced subjective responses and expectancy-related performance effects. In addition, intervention protocols varied markedly across studies, and some outcomes could not be included because raw descriptive data were unavailable. Finally, the GRADE certainty was generally low to very low, reflecting the methodological and reporting limitations of the primary studies. These limitations also constrain the certainty of the meta-analysis itself, because pooled estimates cannot fully compensate for limitations in the underlying evidence base. This means that the current estimates may change as better-designed and more completely reported studies become available. Accordingly, the pooled estimates should be viewed as a quantitative baseline for the current evidence rather than as definitive evidence of efficacy.

### 4.3. Practical Applications and Future Directions

Practically, ammonia inhalation should be used selectively rather than treated as a general performance-enhancing strategy. Its use may be most defensible before brief explosive or repeated high-intensity efforts performed soon after inhalation, particularly when the athlete is already familiar with the stimulus and the task does not require prolonged setup. In contrast, its use before accuracy-based or fine-motor tasks, such as putting or shooting, should be considered with particular care because heightened arousal may be poorly matched to the attentional demands of these skills. Coaches and athletes should also recognize that ammonia inhalants do not appear to consistently improve maximal strength outcomes.

From a practical and regulatory perspective, ammonia inhalation is not listed on the current World Anti-Doping Agency Prohibited List, but this should not be interpreted as formal endorsement of its ergogenic use. Major sport supplement frameworks also do not specifically recommend ammonia inhalation, and some sport-specific organizations restrict or prohibit its use because of safety concerns, including the potential to mask concussion-related symptoms [2]. Therefore, athletes and support staff should check both anti-doping regulations and sport-specific rules before using ammonia inhalants in training or competition. Safety should also be considered, as ammonia is a respiratory and mucosal irritant that may provoke eye, nose, throat, and airway irritation, coughing, dyspnea, wheezing, or chest discomfort, particularly in individuals with asthma, hyperreactive airways, or other respiratory conditions.

Future studies should use adequately powered randomized crossover designs, active sham comparators, standardized administration procedures, and preregistered outcomes. Researchers should report raw means, standard deviations, within-subject correlations, and all relevant time points to facilitate synthesis. Such studies should also directly compare different timing strategies, examine whether sex, training background, habitual ammonia use, and task type modify the response, report adverse events, and assess the effectiveness of blinding given the distinctive odor and irritant properties of ammonia. Given the current uncertainty, more high-quality studies are needed before strong practical recommendations can be made.

## 5. Conclusions

Ammonia inhalation did not significantly improve overall physical performance in the primary analysis, although sensitivity analyses suggested a possible small benefit after removal of influential effects, particularly those involving precision-based tasks. The clearest finding was increased subjective activation/arousal, whereas heart rate and subjective exertion/fatigue showed no consistent effects. Potential benefits may be more likely for explosive or repeated high-intensity tasks than for maximal strength or accuracy-based sport skills. However, the evidence remains limited, and many positive findings were exploratory or sensitivity-based. Further well-controlled studies are needed to clarify when, and for whom, ammonia inhalation may be useful.

## Figures and Tables

**Table 1:** Summary of studies on acute ammonia inhalation and physical performance, perceptual, and physiological responses.

Study; Study Design	Exercise Protocol	Sample; Mean Age (y), Body Weight (kg); Training Status	Dosage of AI and PLA and/or CON; Form;	Administration Protocol; Washout Period	Statistical Significance
Ahn & Ko (2022) [32] N/A Crossover (same trial)	3 m golf putting task	10 M 31.5 ± 3.2, N/A; Professional golfers	AI: Ammonia Sport ampoules, exact dose N/A; CON: no inhalation	AI applied/sprayed to front of face mask for inhalation; Before/during each putting condition via mask; exact timing N/A; 30 min between scent conditions	Performance: Putting success rate: =; HR: =
Barnes et al. (2025) [33] N/A Crossover	IMTP measured at baseline and immediately, 5 min post of 75-min mental-fatigue task	9 M; 23.3 ± 7.4, 82.5 ± 15.7; Resistance-trained	AI: 0.33 mL capsule containing 35% alcohol and 15% ammonia; CON: no AI/room air	Crushed ammonia capsule; AI inhaled 30 s before each IMTP; ≥5 days	Performance: MTP peak force: =, IMTP % change: =
Bartolomei et al. (2018) [5] DB Crossover	CMJ; IMTP maximal isometric strength test	20 M; 26.7 ± 3.7, 80.6 ± 9.0; Resistance-trained	AI: 0.3 mL ammonia capsule; PLA: Vick’s VapoRub; CON: no inhalant	AI and placebo placed in microcentrifuge tubes; Inhaled for 3 s, 10 s before each attempt; tube held 10 cm from upper airways; ≥48 h	Performance: CMJ power: =, IMTP peak force: =;
Campbell et al. (2022) [34] N/A Crossover	Knee-extension and handgrip MVC; CMJ	14 M; 20 ± 1, 76.2 ± 12.7; Recreationally active	AI: 35.99 g Nose Tork ammonium carbonate crystals; Sham: water and cotton wool	Unmarked flask for AI or water sham; Inhaled before each MVC/CMJ repetition; contraction/jump occurred within 30 s; 7 days	Performance: Knee-extension MVC: =, Knee-extension RFD: ↑; Handgrip MVC: =, Handgrip RFD: ↑; CMJ peak power: =, Alertness: ↑; HR: ↑
Gavanda et al. (2025) [35] SB Crossover	Bench press 1-RM test	20 M; 26 ± 5, 90.1 ± 9.3; Resistance-trained	AI: 0.3 mL capsule containing 15% ammonia, 35% alcohol, 50% water; PLA: menthol balm in 2.0 mL tube	AI capsule; placebo Eppendorf tube with menthol balm; 15 s before each 1-RM attempt; 7 days	Performance: Bench press 1-RM: =, MCV: =, Power =; HR: =
Malecek et al. (2023) [36] N/A Crossover (same trial)	Five sessions over 36 h sleep deprivation/recovery; SRT, shooting accuracy, CMJ, and rifle disassembly/reassembly	18 M; 24.1 ± 3.0, 79.3 ± 8.3; Military cadets	AI: 0.3 mL capsule containing 15% ammonia and 35% alcohol; CON: no AI	Crushed ammonia capsule; no-inhalant control; Pre-AI trial; held under nose until withdrawal reflex; 2 min between AI and CON trials	Performance: Shooting accuracy: =, CMJ height: =, Rifle disassembly/reassembly time: =; RPE: ↓; HR: ↑
Perry et al. (2016) [3] N/A Crossover (same trial)	Maximal single MTP performed under control and at immediate, 15 s, 30 s, and 60 s after AI.	15 M; 25 ± 5, 99 ± 16 Resistance-trained	AI: 0.3 mL capsule containing 35% alcohol and 15% ammonia; CON: air/no ammonia	Crushed ammonia capsule; air control; MTP performed immediately, 15 s, 30 s, or 60 s after inhalation; 5 min rest between treatments	Performance: MTP peak force: =, RFD: ↑; HR (within AI group): ↑;
Richmond et al. (2014) [6] DB Crossover	85% 1-RM back squat and bench press to failure	25 M; 21.5 ± 2.2, 93.4 ± 14.2; Resistance-trained	AI: liquid ammonia inhalant, dose N/A; PLA: Vick’s VapoRub	AI liquid and placebo gel contained in microcentrifuge tubes; Inhaled 3 s before test; 2–4 days	Performance: Back squat reps: =, Bench press reps: =
Rogers et al. (2023) [4] SB Crossover	3 × 15 s Wingate anaerobic tests	12 F; 21.4 ± 0.8, 60.4 ± 9.1; Physically active (≥150 min/week)	AI: 0.33 cc ampule, 15% ammonia; CON: ~1 mL water	AI ampule or water placed in unmarked container; Single 3 s inhale, 10 s before each Wingate test; ≥48 h	Performance: Wingate mean power: ↑; Wingate peak power: =; RPE: =; Alertness: ↑; Psyched-up energy: ↑; HR: =
Vigil et al. (2018) [7] DB Crossover	Deadlift 1-RM test	20 total (10 M, 10 F); M: 21 ± 1, 72.5 ± 6.8; F: 22 ± 5, 66.2 ± 8.1; Resistance-trained	AI: 0.33 mL capsule containing ammonia 50 mg/15%, denatured alcohol 35%, water 50%; CON: water	Identical opaque bottles containing crushed AI capsule or water; One maximal inhalation before each 1-RM attempt; lift performed within 15 s; 7 days	Performance: Deadlift 1-RM: =

Notes: AI: ammonia; CON: control group; CMJ: Countermovement jump; F: Female; g: Gram; h: Hours; HR: Heart rate; (I)MTP: (Isometric) mid-thigh pull; kg: Kilogram; M: Male; MCV: mean concentric velocity; MVC: Maximum voluntary contraction; mL: Milliliters; min: Minutes; N/A: Not Available or Not Applicable; PLA: Placebo; POMS: Profile of mood states; RFD: Rate of force development; RPE: Rating of perceived exertion; DB: Double-blind trial; SB: Single-blind trial; s: Seconds; SRT: Simple reaction time; y: years old; ↑: Significantly increase; ↓: Significantly decrease; =: no significant change or difference.

## Data Availability

All data analyzed in this study were obtained from previously published studies, which are cited in the manuscript. No new data were generated for this study.

## Supplementary Materials

The following supporting information can be downloaded at: https://www.mdpi.com/article/10.3390/brainsci16080849/s1. S1: PRISMA 2020 checklist; S2: Summary of effect size calculation procedures; S3: Summary forest plot of aggregated study effects; S4: RoB2 assessment tool for risk of bias; S5: Funnel plot; S6: Power visualization; S7: PEDro assessment; S8: GRADE assessment; S9: Sensitivity analysis for primary results; S10: Leave-one-out sensitivity analysis based on level 3; S11: Moderator analysis after excluding outliers of physical performance.

## References

[1] Bender J.M., Popkin C.A. (2024). Ammonia Inhalants: Use, Misuse, and Role in Sports Performance. Sports Health.

[2] Malecek J., Tufano J.J. (2021). Effects of ammonia inhalants in humans: A review of the current literature regarding the benefits, risks, and efficacy. Strength Cond. J..

[3] Perry B.G., Pritchard H.J., Barnes M.J. (2016). Cerebrovascular, Cardiovascular and Strength Responses to Acute Ammonia Inhalation. Eur. J. Appl. Physiol..

[4] Rogers R.R., Beardsley K.G., Cumbie P.E., Ballmann C.G. (2023). Ammonia Inhalants Enhance Psychophysiological Responses and Performance during Repeated High Intensity Exercise. Res. Q. Exerc. Sport.

[5] Bartolomei S., Nigro F., Gubellini L., Semprini G., Ciacci S., Hoffman J.R., Merni F. (2018). Acute effects of ammonia inhalants on strength and power performance in trained men. J. Strength Cond. Res..

[6] Richmond S., Potts A., Sherman J. (2014). The Impact of Ammonia Inhalants on Strength Performance in Resistance Trained Males. J. Exerc. Physiol. Online.

[7] Vigil J.N., Sabatini P.L., Hill L.C., Swain D.P., Branch J.D. (2018). Ammonia inhalation does not increase deadlift 1-repetition maximum in college-aged male and female weight lifters. J. Strength Cond. Res..

[8] Bezuglov E., Achkasov E., Vakhidov T., Kapralova E., Malyakin G., Lebedenko E., Frolov V., Butovskiy M., Arroyo O.E. (2025). Ammonia Inhalation for Enhancing Physical Performance and Exercise Tolerance in Physically Active Individuals and Athletes: A Systematic Review. Retos.

[9] Page M.J., McKenzie J.E., Bossuyt P.M., Boutron I., Hoffmann T.C., Mulrow C.D., Shamseer L., Tetzlaff J.M., Akl E.A., Brennan S.E. (2021). The PRISMA 2020 Statement: An Updated Guideline for Reporting Systematic Reviews. BMJ.

[10] Premaratne S., Newman J., Hobbs S., Garnham A., Wall M. (2020). Meta-Analysis of Direct Surgical versus Endovascular Revascularization for Aortoiliac Occlusive Disease. J. Vasc. Surg..

[11] Grgic J. (2018). Caffeine Ingestion Enhances Wingate Performance: A Meta-Analysis. Eur. J. Sport Sci..

[12] Sterne J.A.C., Savović J., Page M.J., Elbers R.G., Blencowe N.S., Boutron I., Cates C.J., Cheng H.-Y., Corbett M.S., Eldridge S.M. (2019). RoB 2: A Revised Tool for Assessing Risk of Bias in Randomised Trials. BMJ.

[13] Higgins J., Thomas J., Chandler J., Cumpston M., Li T., Page M.J., Welch V.A. (2019). Cochrane Handbook for Systematic Reviews of Interventions.

[14] Hedges L.V. (1983). A Random Effects Model for Effect Sizes. Psychol. Bull..

[15] Cumpston M., Li T., Page M.J., Chandler J., Welch V.A., Higgins J.P., Thomas J. (2019). Updated Guidance for Trusted Systematic Reviews: A New Edition of the Cochrane Handbook for Systematic Reviews of Interventions. Cochrane Database Syst. Rev..

[16] Deng H., Fan X., Liu P., Song T., Ahmad Fuaad A.A.-H., Bin Mohd Nasiruddin N.J., Bin Naharudin M.N. (2025). Fed, Not Fasted: Is Carbohydrate Mouth Rinsing Still Ergogenic? A Three-Level Meta-Analysis. J. Int. Soc. Sports Nutr..

[17] Assink M., Wibbelink C. (2016). Fitting Three-Level Meta-Analytic Models in R: A Step-by-Step Tutorial. Quant. Methods Psychol..

[18] Kadlec D., Sainani K.L., Nimphius S. (2023). With Great Power Comes Great Responsibility: Common Errors in Meta-Analyses and Meta-Regressions in Strength & Conditioning Research. Sports Med..

[19] Van den Noortgate W., López-López J.A., Marín-Martínez F., Sánchez-Meca J. (2013). Three-Level Meta-Analysis of Dependent Effect Sizes. Behav. Res..

[20] Cheung M.W.-L. (2019). A Guide to Conducting a Meta-Analysis with Non-Independent Effect Sizes. Neuropsychol. Rev..

[21] Pustejovsky J.E., Tipton E. (2022). Meta-Analysis with Robust Variance Estimation: Expanding the Range of Working Models. Prev. Sci..

[22] Nakagawa S., Noble D.W.A., Senior A.M., Lagisz M. (2017). Meta-Evaluation of Meta-Analysis: Ten Appraisal Questions for Biologists. BMC Biol..

[23] Borg D.N., Impellizzeri F.M., Borg S.J., Hutchins K.P., Stewart I.B., Jones T., Baguley B.J., Orssatto L.B.R., Bach A.J.E., Osborne J.O. (2024). Meta-Analysis Prediction Intervals Are under Reported in Sport and Exercise Medicine. Scand. J. Med. Sci. Sports.

[24] IntHout J., Ioannidis J.P.A., Rovers M.M., Goeman J.J. (2016). Plea for Routinely Presenting Prediction Intervals in Meta-Analysis. BMJ Open.

[25] Quintana D.S. (2023). A Guide for Calculating Study-Level Statistical Power for Meta-Analyses. Adv. Methods Pract. Psychol. Sci..

[26] McKay A.K.A., Stellingwerff T., Smith E.S., Martin D.T., Mujika I., Goosey-Tolfrey V.L., Sheppard J., Burke L.M. (2022). Defining Training and Performance Caliber: A Participant Classification Framework. Int. J. Sports Physiol. Perform..

[27] Wickham H. (2011). Ggplot2. WIREs Comput. Stat..

[28] Peters J.L., Sutton A.J., Jones D.R., Abrams K.R., Rushton L. (2008). Contour-Enhanced Meta-Analysis Funnel Plots Help Distinguish Publication Bias from Other Causes of Asymmetry. J. Clin. Epidemiol..

[29] Egger M., Smith G.D., Schneider M., Minder C. (1997). Bias in Meta-Analysis Detected by a Simple, Graphical Test. BMJ.

[30] Sterne J.A.C., Sutton A.J., Ioannidis J.P.A., Terrin N., Jones D.R., Lau J., Carpenter J., Rücker G., Harbord R.M., Schmid C.H. (2011). Recommendations for Examining and Interpreting Funnel Plot Asymmetry in Meta-Analyses of Randomised Controlled Trials. BMJ.

[31] Schünemann H.J., Higgins J.P., Vist G.E., Glasziou P., Akl E.A., Skoetz N., Guyatt G.H. (2019). Completing ‘Summary of Findings’ Tables and Grading the Certainty of the Evidence. Cochrane Handbook for Systematic Reviews of Interventions.

[32] Ahn H., Ko J. (2022). The Effect of Olfactory Inhalation on KPGA Golfers’ Putting Performance, Postural Stability and Heart Rate. Int. J. Environ. Res. Public Health.

[33] Barnes M.J., O’Connor E., van Zanten J. (2025). The Effect of Ammonia Inhalants on Mental-Fatigue-Related Force Loss. J. Funct. Morphol. Kinesiol..

[34] Campbell A.K., Williamson C.E., Macgregor L.J., Hamilton D.L. (2022). Elevated Arousal Following Acute Ammonia Inhalation Is Not Associated with Increased Neuromuscular Performance. Eur. J. Sport Sci..

[35] Gavanda S., Wehner F., Isenmann E., Geisler S., Laborde S., Held S. (2025). The Effects of Acute Ammonia Inhalation on Salivary Cortisol Concentration and Bench Press Performance: A Randomized, Single-Blind Crossover Trial. Sport Sci. Health.

[36] Maleček J., Omcirk D., Skálová K., Pádecký J., Janikov M.T., Obrtel M., Jonáš M., Kolář D., Michalička V., Sýkora K. (2023). Effects of 36 Hours of Sleep Deprivation on Military-Related Tasks: Can Ammonium Inhalants Maintain Performance?. PLoS ONE.

[37] Finger T.E., Böttger B., Hansen A., Anderson K.T., Alimohammadi H., Silver W.L. (2003). Solitary Chemoreceptor Cells in the Nasal Cavity Serve as Sentinels of Respiration. Proc. Natl. Acad. Sci. USA.

[38] Yerkes R.M., Dodson J.D. (1908). The Relation of Strength of Stimulus to Rapidity of Habit-Formation. J. Comp. Neurol. Psychol..

[39] Beilock S.L., Carr T.H. (2001). On the Fragility of Skilled Performance: What Governs Choking under Pressure?. J. Exp. Psychol. Gen..

[40] Easterbrook J.A. (1959). The Effect of Emotion on Cue Utilization and the Organization of Behavior. Psychol. Rev..

[41] Aagaard P., Simonsen E.B., Andersen J.L., Magnusson P., Dyhre-Poulsen P. (2002). Increased Rate of Force Development and Neural Drive of Human Skeletal Muscle Following Resistance Training. J. Appl. Physiol..

[42] Maffiuletti N.A., Aagaard P., Blazevich A.J., Folland J., Tillin N., Duchateau J. (2016). Rate of Force Development: Physiological and Methodological Considerations. Eur. J. Appl. Physiol..

[43] Folland J.P., Williams A.G. (2007). Morphological and Neurological Contributions to Increased Strength. Sports Med..

[44] Suchomel T.J., Nimphius S., Bellon C.R., Stone M.H. (2018). The Importance of Muscular Strength: Training Considerations. Sports Med..

