# Supplementary material for: Smelling Salts or Smoke and Mirrors? Acute Ammonia Inhalation, Arousal, and Physical Performance: A Systematic Review and Meta-Analysis
Source: Brain Sci. 2026 Aug 10;16(8):849. doi: 10.3390/brainsci16080849 (PMC13511091; doi:10.3390/brainsci16080849)
Supplement: Supplementary file 1 [file brainsci-16-00849-s001.zip › brainsci-4437452-supplementary.pdf]

Electronic Supplementary Material Contents

Number	Material	Page
1	Electronic Supplementary Material S1 (PRISMA 2020 Checklist)	P2-5
2	Electronic Supplementary Material S2 (Table. Summary of Effect Size Calculation Procedures)	P6-7
3	Electronic Supplementary Material S3 (Summary Forest Plot of Aggregated Study Effects)	P8-11
4	Electronic Supplementary Material S4 (RoB2 Assessment Tool for Risk of Bias)	P12
5	Electronic Supplementary Material S5 (Funnel Plot)	P13-14
6	Electronic Supplementary Material S6 (Power Visualization)	P15-16
7	Electronic Supplementary Material S7 (PEDro Assessment)	P17
8	Electronic Supplementary Material S8 (GRADE Assessment)	P18-20
9	Electronic Supplementary Material S9 (Sensitivity Analysis for Primary Results)	P21-22
10	Electronic Supplementary Material S10 (Leave-one-out Sensitivity Analysis based on Level-3)	P23-24
11	Electronic Supplementary Material S11 (Moderator Analysis After Excluding Outlier of Physical Performance)	P25

Electronic Supplementary Material S1 (PRISMA 2020 Checklist)

Section and Topic	Item #	Checklist item	Location where item is reported (on page)
**TITLE: Smelling Salts or Smoke and Mirrors? Acute Ammonia Inhalation, Arousal, and Physical Performance: A Systematic Review and Meta-Analysis**			
Title	1	Identify the report as a systematic review.	P1
**ABSTRACT**			
Abstract	2	See the PRISMA 2020 for Abstracts checklist.	P1-2
**INTRODUCTION**			
Rationale	3	Describe the rationale for the review in the context of existing knowledge.	P2-3
Objectives	4	Provide an explicit statement of the objective(s) or question(s) the review addresses.	P2-3
**METHODS**			
Eligibility criteria	5	Specify the inclusion and exclusion criteria for the review and how studies were grouped for the syntheses.	P3
Information sources	6	Specify all databases, registers, websites, organisations, reference lists and other sources searched or consulted to identify studies. Specify the date when each source was last searched or consulted.	P3-4
Search strategy	7	Present the full search strategies for all databases, registers and websites, including any filters and limits used.	P3-4
Selection process	8	Specify the methods used to decide whether a study met the inclusion criteria of the review, including how many reviewers screened each record and each report retrieved, whether they worked independently, and if applicable, details of automation tools used in the process.	P4
Data collection process	9	Specify the methods used to collect data from reports, including how many reviewers collected data from each report, whether they worked independently, any processes for obtaining or confirming data from study investigators, and if applicable, details of automation tools used in the process.	P4
Data items	10a	List and define all outcomes for which data were sought. Specify whether all results that were compatible with each outcome domain in each study were sought (e.g. for all measures, time points, analyses), and if not, the methods used to decide which results to collect.	P4
	10b	List and define all other variables for which data were sought (e.g. participant and intervention characteristics, funding sources). Describe any assumptions made about any missing or unclear information.	P4

Section and Topic	Item #	Checklist item	Location where item is reported (on page)
Study risk of bias assessment	11	Specify the methods used to assess risk of bias in the included studies, including details of the tool(s) used, how many reviewers assessed each study and whether they worked independently, and if applicable, details of automation tools used in the process.	P4
Effect measures	12	Specify for each outcome the effect measure(s) (e.g. risk ratio, mean difference) used in the synthesis or presentation of results.	P4-5
Synthesis methods	13a	Describe the processes used to decide which studies were eligible for each synthesis (e.g. tabulating the study intervention characteristics and comparing against the planned groups for each synthesis (item #5)).	P5
	13b	Describe any methods required to prepare the data for presentation or synthesis, such as handling of missing summary statistics, or data conversions.	P5
	13c	Describe any methods used to tabulate or visually display results of individual studies and syntheses.	P5-6
	13d	Describe any methods used to synthesize results and provide a rationale for the choice(s). If meta-analysis was performed, describe the model(s), method(s) to identify the presence and extent of statistical heterogeneity, and software package(s) used.	P5
	13e	Describe any methods used to explore possible causes of heterogeneity among study results (e.g. subgroup analysis, meta-regression).	P5-6
	13f	Describe any sensitivity analyses conducted to assess robustness of the synthesized results.	P6
Reporting bias assessment	14	Describe any methods used to assess risk of bias due to missing results in a synthesis (arising from reporting biases).	P6
Certainty assessment	15	Describe any methods used to assess certainty (or confidence) in the body of evidence for an outcome.	P6
**RESULTS**			
Study selection	16a	Describe the results of the search and selection process, from the number of records identified in the search to the number of studies included in the review, ideally using a flow diagram.	P6-7
	16b	Cite studies that might appear to meet the inclusion criteria, but which were excluded, and explain why they were excluded.	P6-7
Study characteristics	17	Cite each included study and present its characteristics.	P7-9
Risk of bias in	18	Present assessments of risk of bias for each included study.	P11-12

Section and Topic	Item #	Checklist item	Location where item is reported (on page)
studies			
Results of individual studies	19	For all outcomes, present, for each study: (a) summary statistics for each group (where appropriate) and (b) an effect estimate and its precision (e.g. confidence/credible interval), ideally using structured tables or plots.	P9-10
Results of syntheses	20a	For each synthesis, briefly summarise the characteristics and risk of bias among contributing studies.	P11-12
	20b	Present results of all statistical syntheses conducted. If meta-analysis was done, present for each the summary estimate and its precision (e.g. confidence/credible interval) and measures of statistical heterogeneity. If comparing groups, describe the direction of the effect.	P9-10
	20c	Present results of all investigations of possible causes of heterogeneity among study results.	P10-11
	20d	Present results of all sensitivity analyses conducted to assess the robustness of the synthesized results.	P12-13
Reporting biases	21	Present assessments of risk of bias due to missing results (arising from reporting biases) for each synthesis assessed.	P11-12
Certainty of evidence	22	Present assessments of certainty (or confidence) in the body of evidence for each outcome assessed.	P12
**DISCUSSION**			
Discussion	23a	Provide a general interpretation of the results in the context of other evidence.	P13-15
	23b	Discuss any limitations of the evidence included in the review.	P15-16
	23c	Discuss any limitations of the review processes used.	P15-16
	23d	Discuss implications of the results for practice, policy, and future research.	P16
**OTHER INFORMATION**			
Registration and protocol	24a	Provide registration information for the review, including register name and registration number, or state that the review was not registered.	P3
	24b	Indicate where the review protocol can be accessed, or state that a protocol was not prepared.	P16-17
	24c	Describe and explain any amendments to information provided at registration or in the protocol.	N/A
Support	25	Describe sources of financial or non-financial support for the review, and the role of the funders or sponsors in the review.	P16-17
Competing	26	Declare any competing interests of review authors.	P16-17

Section and Topic	Item #	Checklist item	Location where item is reported (on page)
interests			
Availability of data, code and other materials	27	Report which of the following are publicly available and where they can be found: template data collection forms; data extracted from included studies; data used for all analyses; analytic code; any other materials used in the review.	N/A

Electronic Supplementary Material S2 (Table. Summary of Effect Size Calculation Procedures)

Step	Data condition	Calculation Formula	Description
①	Pre–post data available	$\Delta AI = M_{AIpost} - M_{AIpre}$ $\Delta PLA = M_{PLApost} - M_{PLApre}$ $MD = \Delta AI - \Delta PLA$	Calculate the mean change within each condition (AI and placebo) and use their difference (MD) as the comparison metric.
②	Only post-intervention data available	$MD = M_{AI} - M_{PLA}$	When only post values are reported, the mean difference between conditions is used directly.
③	SD of change (for pre–post data)	$SD_{change} = \sqrt{SD^2{}_{pre} + SD^2{}_{post} - (2 \times r \times SD_{pre} \times SD_{post})}$	Computes the within-subject SD of change using the correlation (r) between pre- and post-measurements.
④	Pooled SD for (*i*) crossover and (*ii*) parallel design	$SD_{pooled} = \sqrt{\frac{SD^2{}_{AI} + SD^2{}_{PLA}}{2}}$ (*i*) $SD_{pooled} = \sqrt{\frac{(n_{AI}-1) \times SD^2_{AI} + (n_{PLA}-1) \times SD^2_{PLA}}{n_{AI}+n_{PLA}-2}}$ (*ii*)	Estimates the pooled variability across both conditions.
⑤		$Hedges' \ g = \frac{M_{AI} - M_{PLA}}{SD_{pooled}} \times \left(1 - \frac{3}{4(N-1)-1}\right)$ (*i*)	Standardized mean difference corrected for small-sample bias.

	Effect size (Hedges' *g*) for (*i*) crossover and (*ii*) parallel design	$\text{Hedges' g} = \frac{M_{AI} - M_{PLA}}{SD_{pooled}} \times \left(1 - \frac{3}{4(n_{AI}+n_{PLA}-2)-1}\right)$ (*ii*)	
⑥	Standard error (SE) of *g* for (*i*) crossover and (*ii*) parallel design	$SE = \sqrt{\frac{1}{N} + \frac{g^2}{2N}} \times \sqrt{2(1-r)}$ (i)	(*i*) Adjusts SE to account for within-subject correlation between AI and placebo conditions. (*ii*) Calculates SE from independent AI and placebo group sizes, with the harmonic mean accounting for potential imbalance.
		$SE = \sqrt{\frac{n_{AI}+n_{PLA}}{n_{AI} \times n_{PLA}} + \frac{g^2}{2(n_{AI}+n_{PLA})}}$ (*ii*)	
⑦	Assumed correlation (*r*)	*r* = 0.50 (primary analysis)<br>*r* = 0.20 and 0.80 (sensitivity)	The same r value was used for both pre - post and between-condition comparisons to ensure consistency and comparability.

Electronic Supplementary Material S3 (Summary Forest Plot of Aggregated Study Effects)

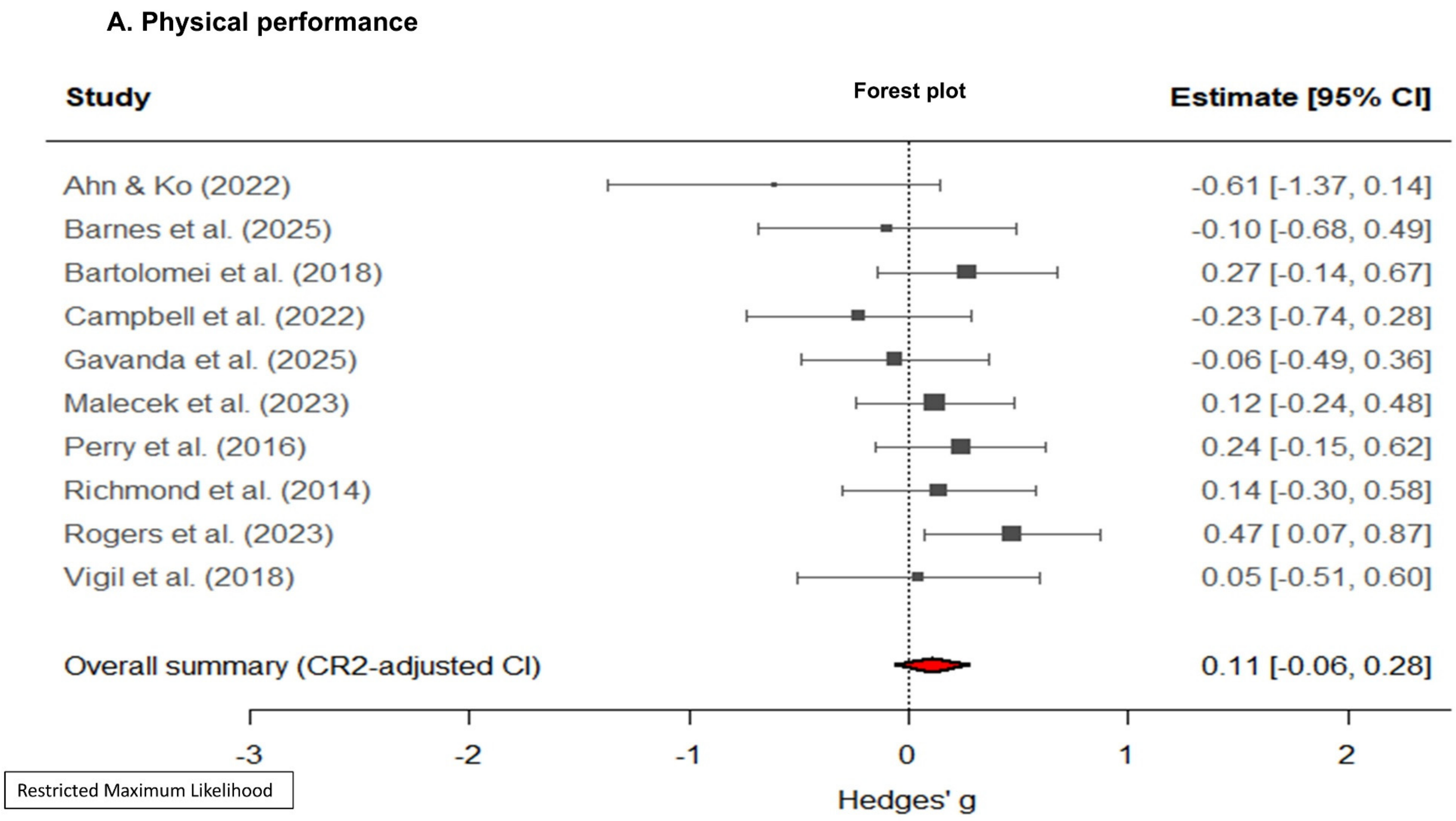

B. Heart rate

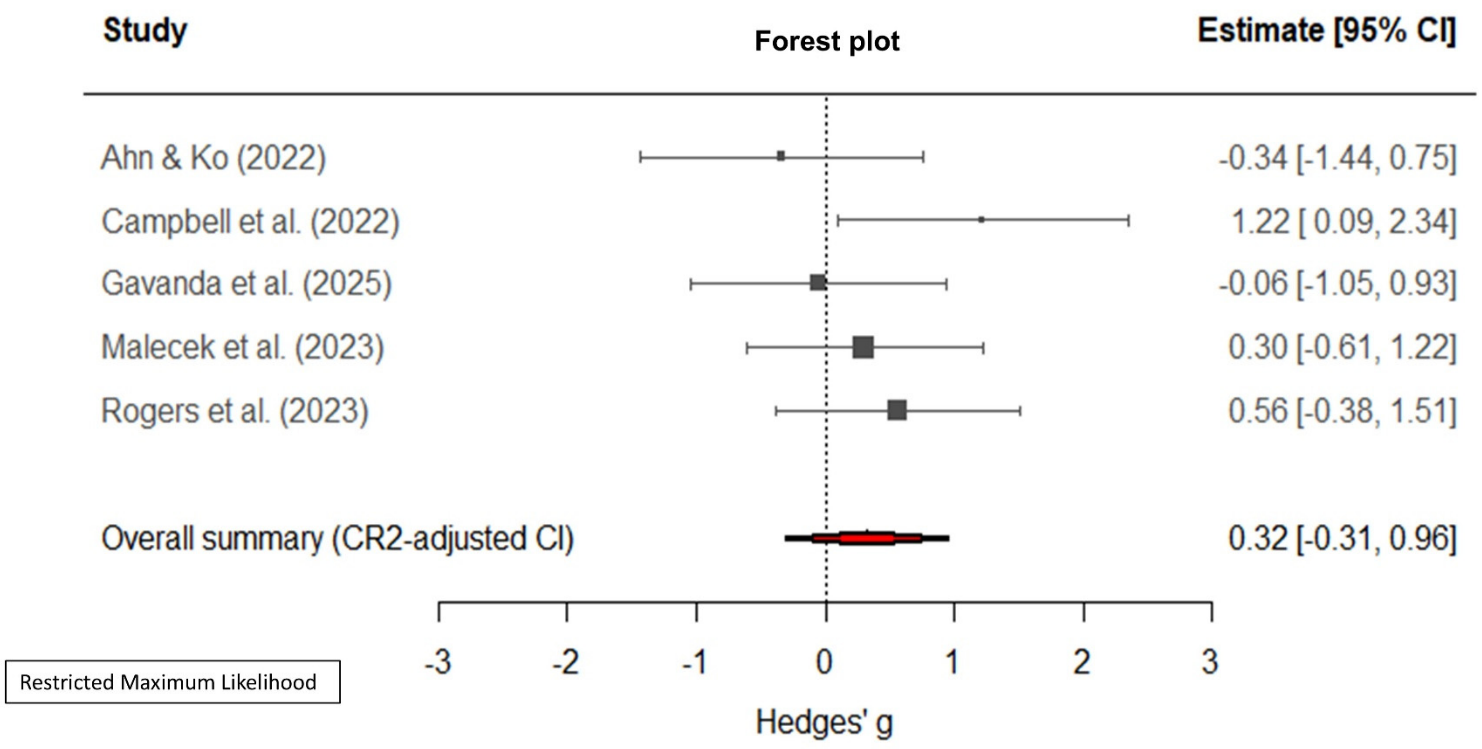
Study
Forest plot
Estimate [95% CI]
Ahn & Ko (2022)
-0.34 [-1.44, 0.75]
Campbell et al. (2022)
1.22 [0.09, 2.34]
Gavanda et al. (2025)
-0.06 [-1.05, 0.93]
Malecek et al. (2023)
0.30 [-0.61, 1.22]
Rogers et al. (2023)
0.56 [-0.38, 1.51]
Overall summary (CR2-adjusted CI)
0.32 [-0.31, 0.96]
Restricted Maximum Likelihood
-3
-2
-1
0
1
2
3
Hedges' g

C. Subjective exertion/fatigue

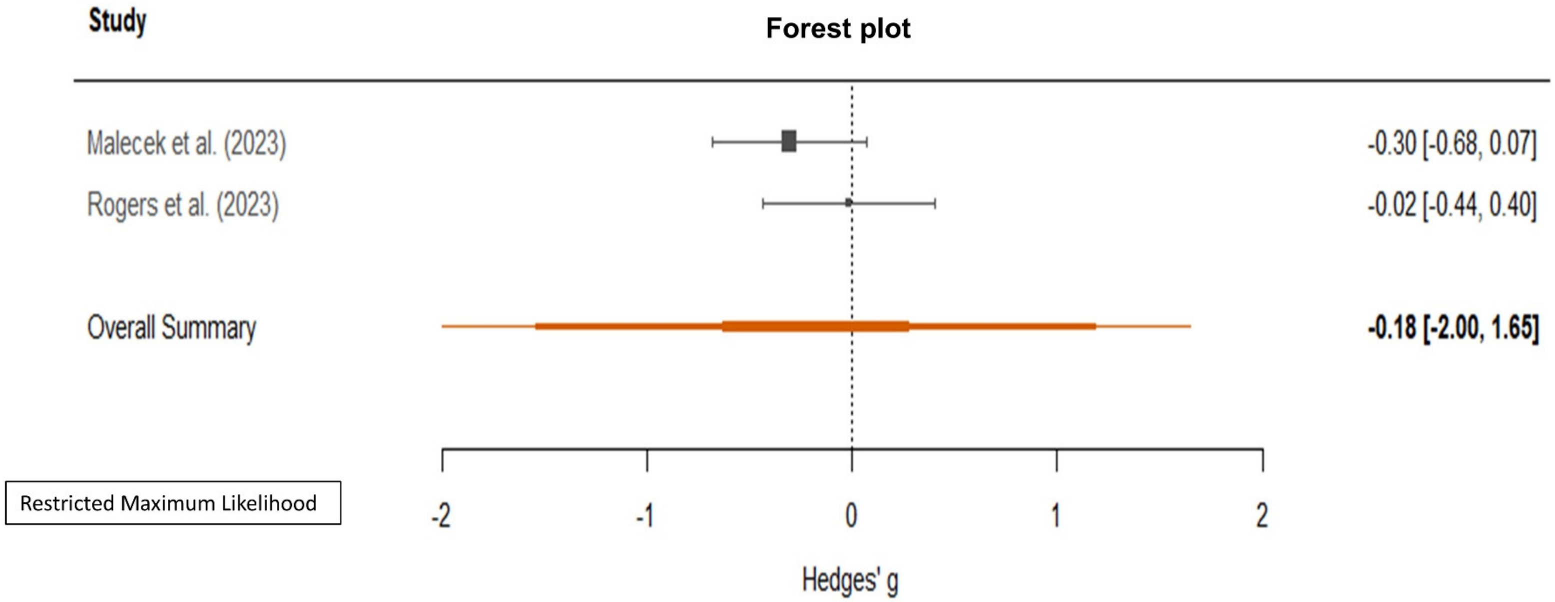

D. Subjective activation/arousal

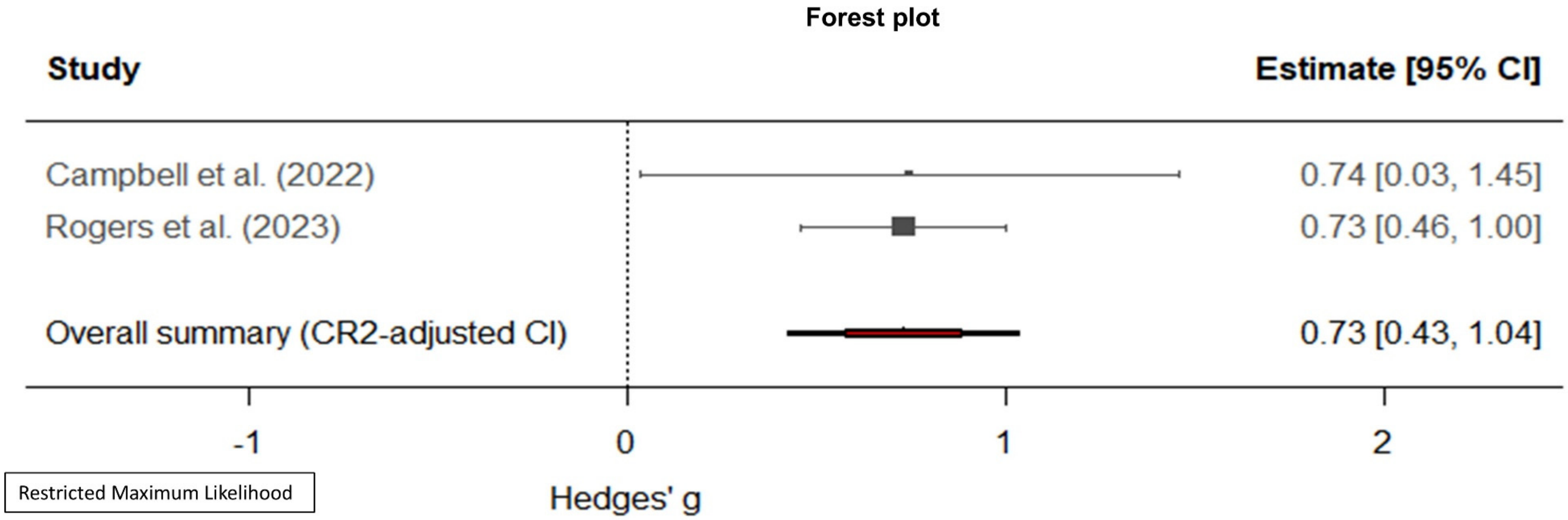

Notes: Hedges' g, the effect size indicators used in the pooled; ***95%CI***, 95% confidence interval after CR2 small-sample correction; ***The size of each black square***, represents the relative weight of the study in the meta-analysis; ***A***, for physical performance; ***B***, for heart rate; ***C,*** for subjective exertion/fatigue; ***D,*** for subjective activation/arousal.

Electronic Supplementary Material S4 (RoB2 Assessment Tool for Risk of Bias)

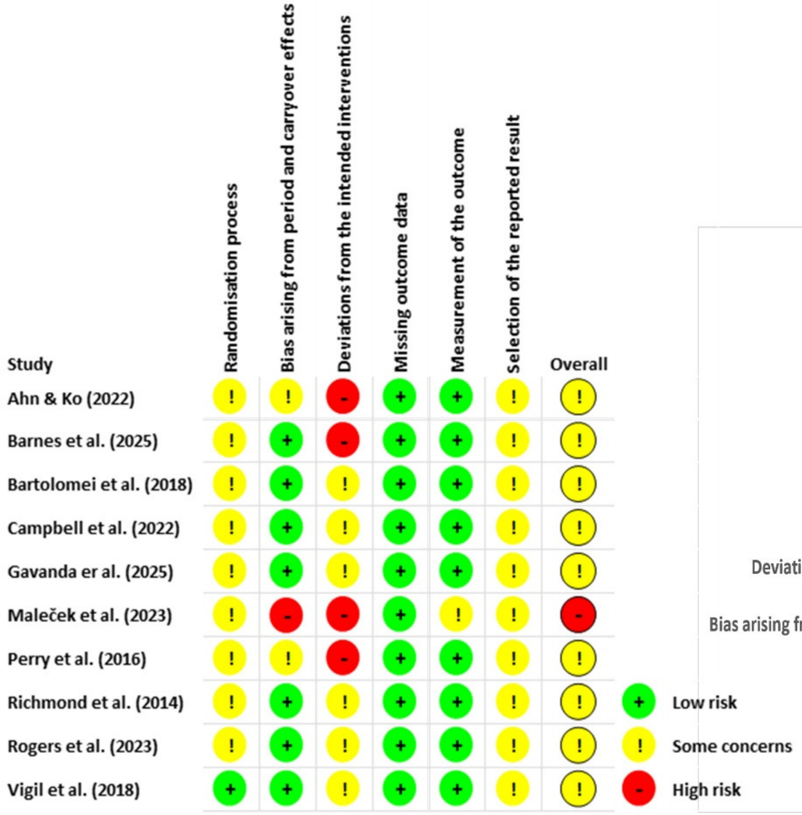

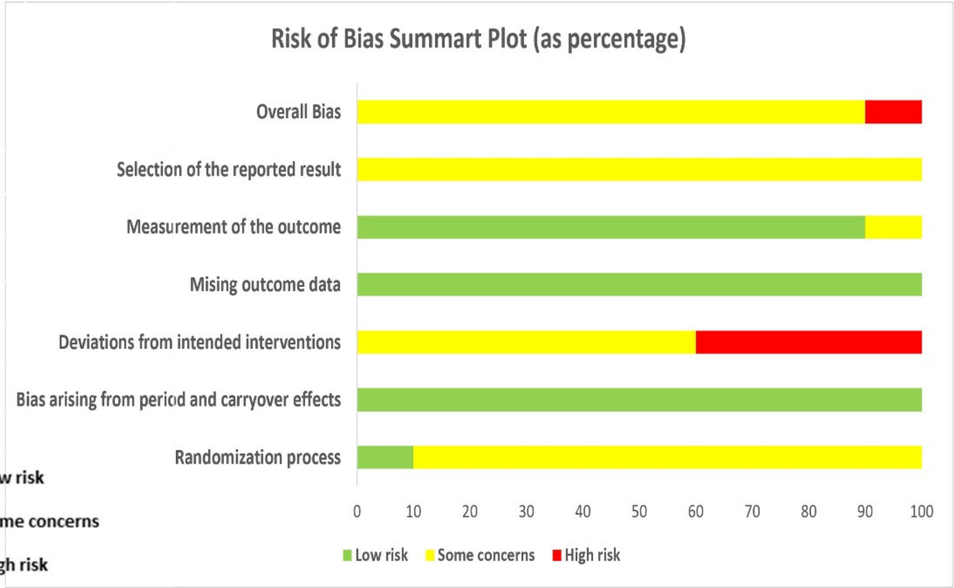

.

Electronic Supplementary Material S5 (Funnel Plot)

Primary outcomes:

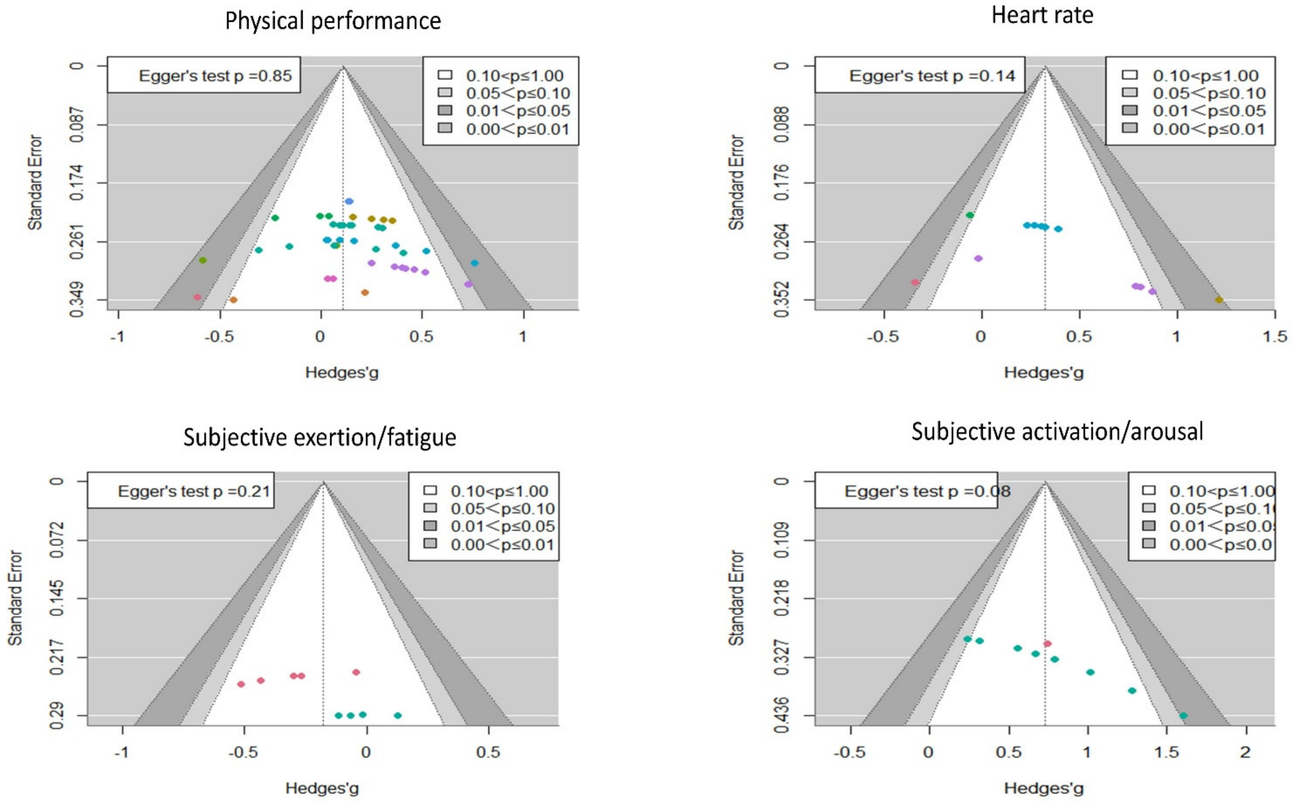

Notes: Colorful circles, represent different effect size points.

Moderator outcomes:

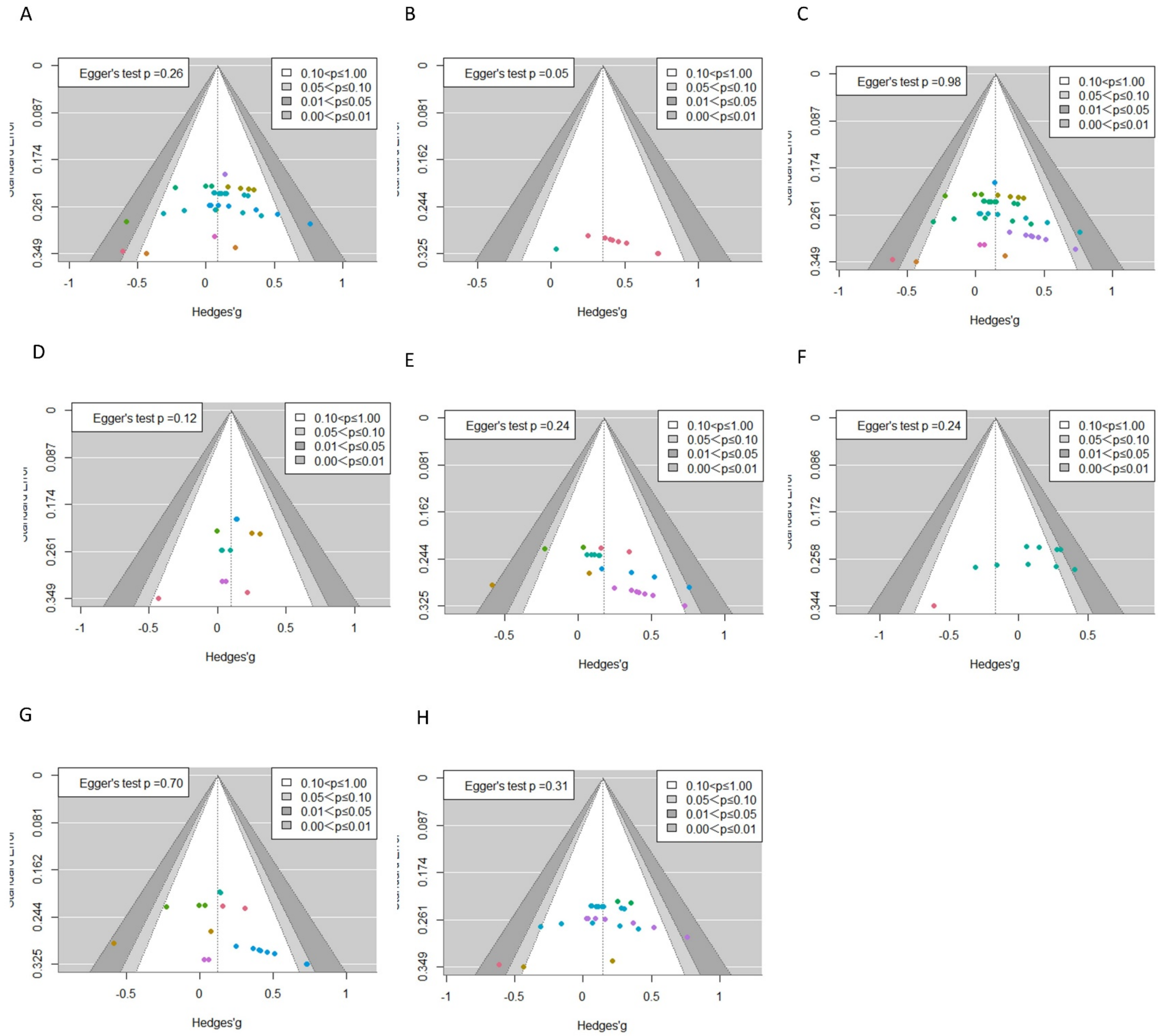

Notes: Colorful circles, represent different effect size points From ***A*** to ***H***, they are male, female, trained, Strength performance, Power/explosive performance, Sport- or task-specific performance, Sham inhalation, No-inhalation.

Electronic Supplementary Material S6 (Power Visualization)

Primary outcomes:

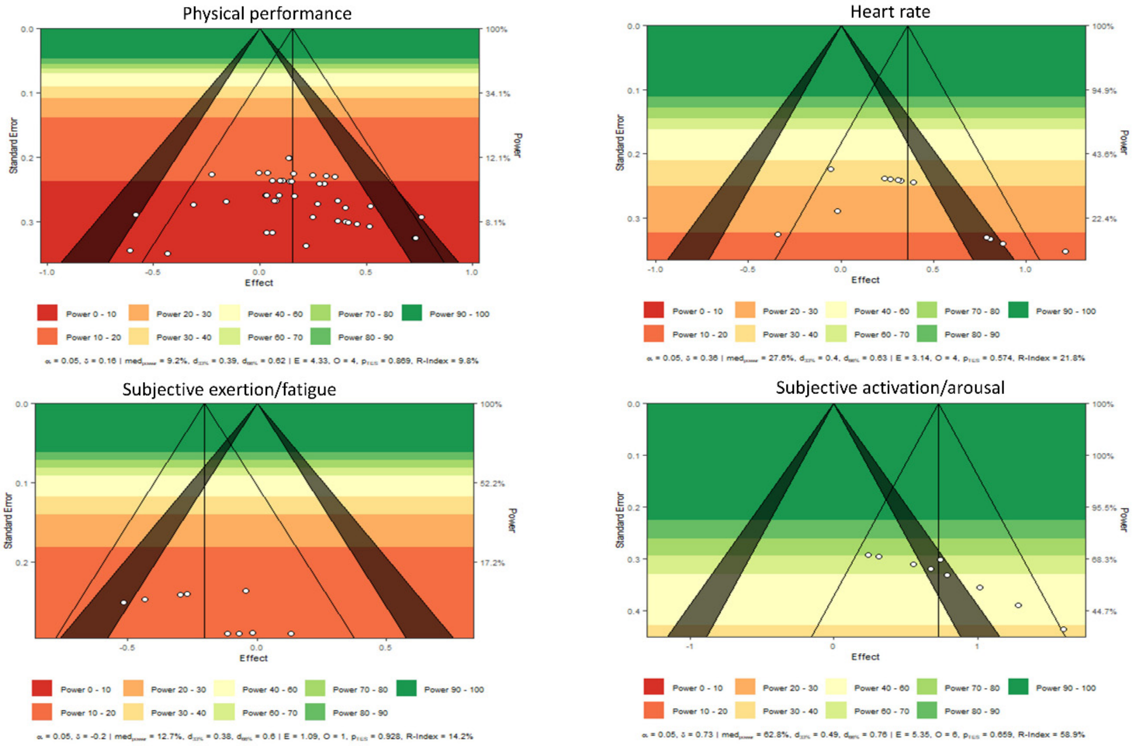

Notes**:** The vertical solid line represents the pooled effect size, and the vertical dash line represents the adjusted pooled effect size. Significance contours at .05 and .01 levels are noted by the shaded area. ***manpower*** indicates the median power of all included effect sizes. ***d33%*** and ***d66%*** indicate the true effect sizes necessary for achieving 33% and 66% levels of median power. ***E, O, and PTES*** show the results of a test of excess significance. ***R-index*** denotes the expected replicability of findings.

Moderator outcomes:

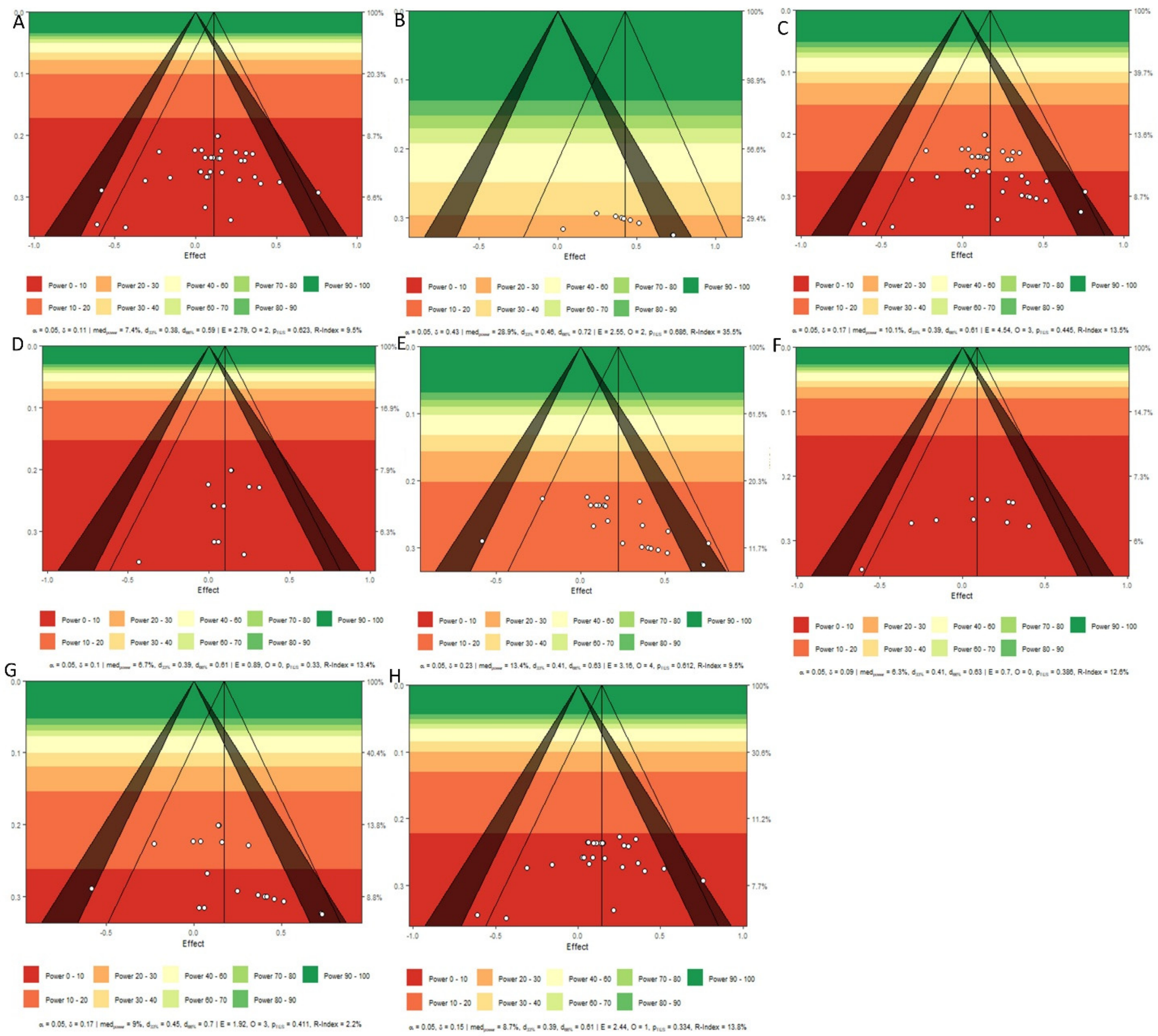

Notes: The vertical solid line represents the pooled effect size, and the vertical dash line represents the adjusted pooled effect size. Significance contours at .05 and .01 levels are noted by the shaded area. ***manpower*** indicates the median power of all included effect sizes. ***d33%*** and ***d66%*** indicate the true effect sizes necessary for achieving 33% and 66% levels of median power. ***E, O, and PTES*** show the results of a test of excess significance. ***R-index*** denotes the expected replicability of findings. From ***A*** to ***H***, they are male, female, trained, Strength performance, Power/explosive performance, Sport- or task-specific performance, Sham inhalation, No-inhalation.

Electronic Supplementary Material S7 (PEDro Assessment)

Table A. PEDro assessment results of exercise performance researches

Study	Item 1	Item 2	Item 3	Item 4	Item 5	Item 6	Item 7	Item 8	Item 9	Item 10	Item 11	Item 12	Total
Ahn & Ko (2022)	1	0	0	0	0	0	0	1	1	1	1	0	4
Barnes et al. (2025)	1	1	0	1	0	0	0	1	1	1	1	0	6
Bartolomei et al. (2018)	1	1	0	1	1	1	1	1	1	1	1	0	9
Campbell et al. (2022)	1	1	0	1	0	0	0	1	1	1	1	0	6
Gavanda et al. (2025)	1	1	0	1	1	0	1	1	1	1	1	0	8
Malecek et al. (2023)	1	1	0	1	0	0	0	1	1	1	1	0	6
Perry et al. (2016)	1	1	0	1	0	0	0	1	1	1	1	0	6
Richmond et al. (2014)	1	1	0	1	1	1	0	1	1	1	1	0	8
Rogers et al. (2023)	1	1	0	1	0	0	0	1	1	1	1	0	6
Vigil et al. (2018)	1	1	1	1	0	0	0	1	1	1	1	0	7

Electronic Supplementary Material S8 (GRADE Assessment)

Outcome	K	Certainty of Evidence Assessment					Hedges' g [95% CI] *	GRADE†
		Risk of Bias	Inconsistency	Indirectness	Imprecision	Others		
**Primary Outcome**								
Physical performance	K = 47	Serious	Serious	Not serious	Serious	None	0.11 [−0.06, 0.28]	⊕◯◯◯ Very low
Heart rate	K = 12	Serious	Serious	Not serious	Serious	None	0.32 [−0.31, 0.96]	⊕◯◯◯ Very low
Subjective exertion/fatigue	K = 9	Serious	Not serious	Not serious	Very serious	None	−0.17 [−2.00, 1.65]	⊕◯◯◯ Very low
Subjective activation/arousal	K = 9	Not serious	Not serious	Not serious	Very serious	None	0.73 [0.43, 1.04] *	⊕⊕◯◯ Low
**Sex**								
Male	K = 38	Serious	Not serious	Serious	Serious	None	0.08 [−0.06,0.23]	⊕◯◯◯ Very low

Female	K = 9	Not serious	Not serious	Not serious	Very serious	None	0.40 [−1.77,2.56]	⊕⊕◯◯ Low
**Training Level**								
Trained	K = 45	Serious	Not serious	Serious	Serious	None	0.15 [−0.02,0.31]	⊕◯◯◯ Very low
Untrained	K = 2	Not serious	Not serious	Not serious	Extremely serious	None	−0.23 [−0.72,0.27]	⊕◯◯◯ Very low
**Performance Type**								
Strength performance	K = 13	Not serious	Not serious	Serious	Serious	None	0.05 [−0.13,0.23]	⊕⊕◯◯ Low
Power/explosive performance	K = 23	Serious	Serious	Not serious	Serious	None	0.16 [−0.13,0.45]	⊕◯◯◯ Very low
Sport- or task-specific performance	K = 11	Serious	Serious	Serious	Very serious	None	0.10 [−1.20,1.40]	⊕◯◯◯ Very low
**Comparator Type**								
Sham inhalation	K = 19	Not serious	Serious	Serious	Serious	None	0.11 [−0.15,0.37]	⊕◯◯◯ Very low

No-inhalation	K = 28	Serious	Not serious	Not serious	Serious	None	0.10 [−0.16,0.36]	⊕⊕◯◯ Low

Notes: K*:** the total number of effects included in the pooled effect size; ***Publication bias, represented by Egger test $p < 0.05$.

***:** The effect size (*Hedges'g*) was significant ($p < 0.05$).

† **GRADE Criteria for Certainty of Evidence**:

High: Very confident in the estimated effect.

Moderate: Moderately confident in the estimated effect.

Low: Limited confidence in the estimated effect.

Very low: Very limited confidence in the estimated effect.

Downgrading criteria: (1) Risk of bias was downgraded when a substantial proportion of contributing studies had risk-of-bias concerns. **(2)** Inconsistency was downgraded when heterogeneity was substantial or when prediction intervals suggested important variability in true effects. **(3)** Indirectness was downgraded when the population, intervention, comparator, or outcome had limited applicability to the main review question. **(4)** Imprecision was downgraded when confidence intervals were wide, crossed the null or clinically relevant thresholds, or when evidence was sparse. **(5)** Publication bias was downgraded when funnel-plot inspection or Egger's test suggested small-study effects or publication bias, where assessment was possible. For subgroup analyses, sparse evidence was considered primarily within the imprecision domain, while other GRADE domains were judged where applicable. Certainty ratings for sparse subgroups were reported for transparency and should be interpreted cautiously and mainly for descriptive purposes, rather than as precise or definitive certainty estimates for subgroup-specific.

Electronic Supplementary Material S9 (Sensitivity Analysis for Primary Results)

Test Methods	K	Hedges' g	95% CI	P	PI	I^2	Power
**Physical performance**							
r = 0.8	47	0.07	[−0.12, 0.26]	0.42	[−0.46, 0.59]	70%	17%
r = 0.2	47	0.14	[−0.02, 0.30]	0.08	[−0.13, 0.41]	11%	56%
ML	47	0.12	[−0.05, 0.28]	0.14	[−0.22, 0.45]	25%	45%
Remove outliers	42	0.21	[0.07, 0.35]	0.01	[−0.01, 0.43]	12%	80%
**Heart rate**							
r = 0.8	12	0.34	[−0.34, 1.02]	0.24	[−0.97, 1.65]	91%	29%
r = 0.2	12	0.32	[−0.22, 0.87]	0.17	[−0.51, 1.16]	47%	37%
ML	12	0.32	[−0.27, 0.92]	0.20	[−0.57, 1.22]	63%	72%
Remove outliers	10	0.30	[−0.38, 0.98]	0.19	[−0.30, 0.90]	40%	59%
**Subjective exertion/fatigue**							
r = 0.8	9	-0.17	[−2.01, 1.67]	0.45	[−0.71, 0.38]	56%	32%
r = 0.2	9	-0.19	[−2.00, 1.62]	0.41	[−0.62, 0.25]	12%	18%
ML	9	-0.19	[−1.98, 1.59]	0.40	[−0.45, 0.06]	4%	29%
Remove outliers	8	-0.22	[−1.69, 1.25]	0.31	[−0.57, 0.13]	12%	31%
**Subjective activation/arousal**							
r = 0.8	9	0.77	[0.48, 1.08]	0.02	[−0.11, 1.66]	74%	90%
r = 0.2	9	0.71	[0.42, 1.00]	0.02	[0.39, 1.03]	0	87%

ML	9	0.72	[0.42, 1.02]	0.02	[0.29, 1.15]	15%	89%
Remove outliers	N/A						

Notes: K, the total number of effects included in the pooled effect size; ***Hedges'g***, the effect size indicators used in the pooled; ***95%CI***, 95% confidence interval after CR2 small-sample correction; ***PI***, Prediction interval, ***P-value***, statistically significant P values for pooled results after CR2 small-sample correction; I^2, quantitative indicators of heterogeneity; ***Power***, exploratory post hoc statistical power estimate for pooled effect size; ***r***, correlation coefficient between menthol group and placebo group; ***ML***, maximum likelihood estimation.

Electronic Supplementary Material S10 (Leave-one-out Sensitivity Analysis based on Level-3)

Physical performance:

A

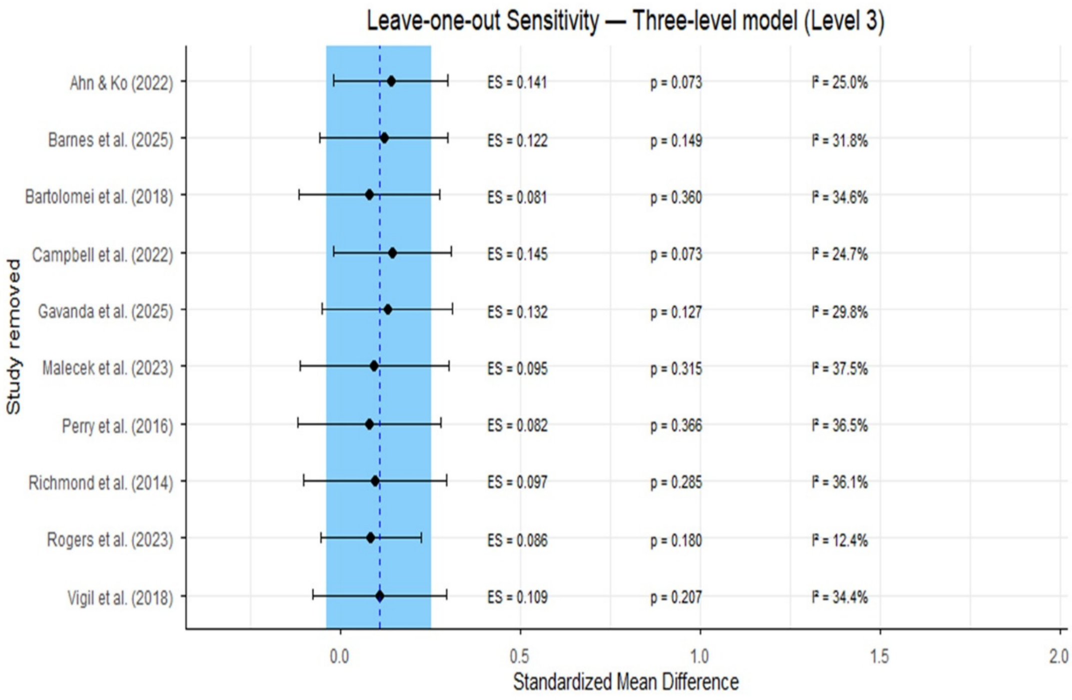

Heart rate:

B

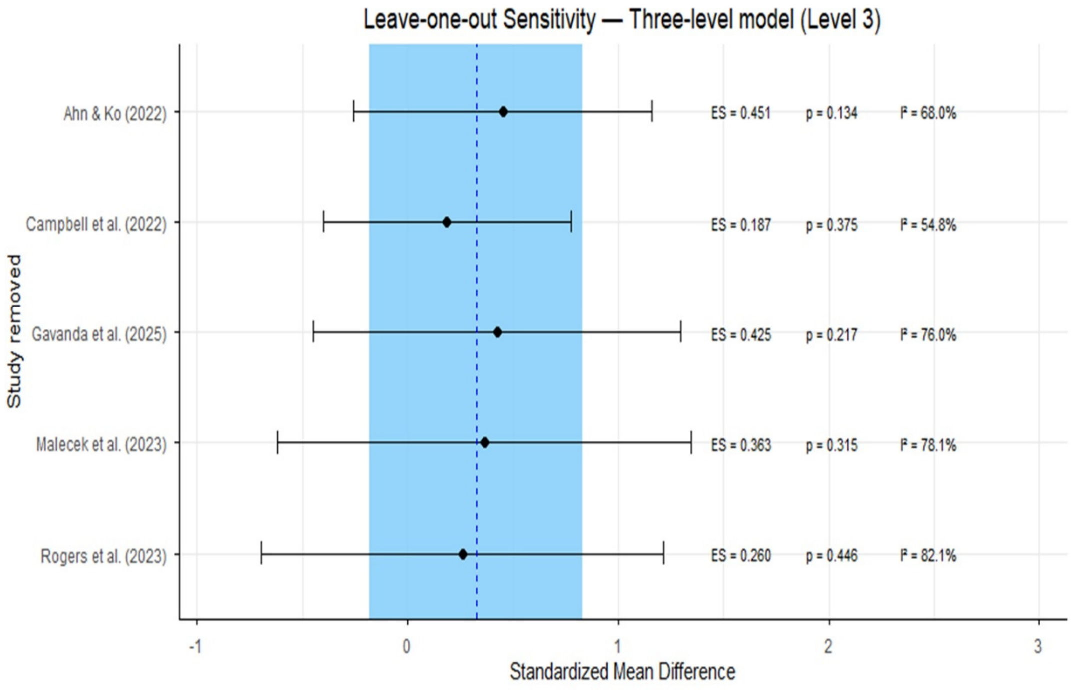

Leave-one-out Sensitivity — Three-level model (Level 3)
Study removed
Ahn & Ko (2022)
Campbell et al. (2022)
Gavanda et al. (2025)
Malecek et al. (2023)
Rogers et al. (2023)
ES = 0.451 p = 0.134 I^2 = 68.0%
ES = 0.187 p = 0.375 I^2 = 54.8%
ES = 0.425 p = 0.217 I^2 = 76.0%
ES = 0.363 p = 0.315 I^2 = 78.1%
ES = 0.260 p = 0.446 I^2 = 82.1%
-1
0
1
2
3
Standardized Mean Difference

Electronic Supplementary Material S11 (Moderator Analysis After Excluding Outlier of Physical performance)

Subgroup	k	g [95% CI]	P_{within}	Forest plot	I^2	$P_{between}$
**Sex**					**0**	**0.01**
Male * †	34	0.15 [0.04, 0.26]	0.02			
Female * †	8	0.47 [0.34, 0.60]	0.01			
**Training Level**					**N/A**	**N/A**
Trained	45	0.14 [-0.02, 0.31]	0.07			
**Performance Type**					**0.09**	**0.27**
Strength performance	13	0.09 [-0.04, 0.23]	0.13			
Power/explosive performance *	20	0.25 [0.02, 0.49]	0.04			
Sport- or task-specific performance	10	0.18 [-0.42, 0.78]	0.22			
**Comparator Type**					**0**	**0.98**
Sham inhalation	17	0.18 [-0.13, 0.49]	0.18			
No-inhalation	25	0.20 [-0.02, 0.42]	0.06			

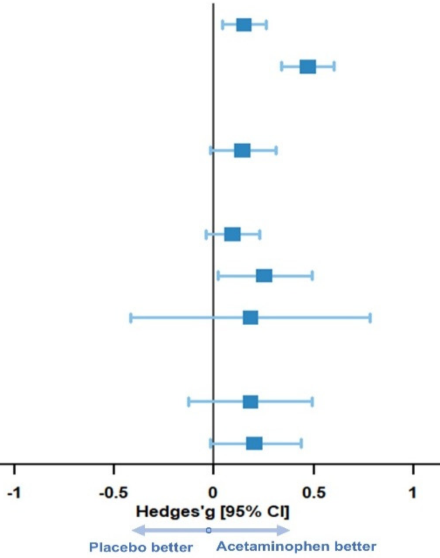

Three-level Moderator Analysis
Restricted Maximum Likelihood

Notes: K, the total number of effects included in the pooled effect size; ***Hedges' g***, the effect size indicators used in the pooled; ***95%CI***, 95% confidence interval; $\boldsymbol{P_{between}}$, CR2-adjusted P value for subgroup differences; $\boldsymbol{P_{within}}$, CR2-adjusted P value for the pooled effect within each subgroup; $\boldsymbol{I^2}$, quantitative indicators of heterogeneity; *****, represents significant differences within category. ($p < 0.05$); †, represents significant differences between moderator. ($p < 0.05$).
